# Brain Cytochrome P450: Navigating Neurological Health and Metabolic Regulation

**DOI:** 10.3390/jox15020044

**Published:** 2025-03-14

**Authors:** Pradeepraj Durairaj, Zixiang Leonardo Liu

**Affiliations:** 1 Department of Chemical and Biomedical Engineering, Florida State University, Tallahassee, FL 32310, USA; 2 Department of Chemical and Biomedical Engineering, Florida A&M University, Tallahassee, FL 32310, USA; 3 Institute for Successful Longevity, Florida State University, Tallahassee, FL 32310, USA

**Keywords:** cytochrome P450, xenobiotic-metabolizing enzymes, brain, neurological health, neurochemistry, neurodegeneration, neurological disorders, neuropathological biomarkers

## Abstract

Human cytochrome P450 (CYP) enzymes in the brain represent a crucial frontier in neuroscience, with far-reaching implications for drug detoxification, cellular metabolism, and the progression of neurodegenerative diseases. The brain’s complex architecture, composed of interconnected cell types and receptors, drives unique neuronal signaling pathways, modulates enzyme functions, and leads to distinct CYP gene expression and regulation patterns compared to the liver. Despite their relatively low levels of expression, brain CYPs exert significant influence on drug responses, neurotoxin susceptibility, behavior, and neurological disease risk. These enzymes are essential for maintaining brain homeostasis, mediating cholesterol turnover, and synthesizing and metabolizing neurochemicals, neurosteroids, and neurotransmitters. Moreover, they are key participants in oxidative stress responses, neuroprotection, and the regulation of inflammation. In addition to their roles in metabolizing psychotropic drugs, substances of abuse, and endogenous compounds, brain CYPs impact drug efficacy, safety, and resistance, underscoring their importance beyond traditional drug metabolism. Their involvement in critical physiological processes also links them to neuroprotection, with significant implications for the onset and progression of neurodegenerative diseases. Understanding the roles of cerebral CYP enzymes is vital for advancing neuroprotective strategies, personalizing treatments for brain disorders, and developing CNS-targeting therapeutics. This review explores the emerging roles of CYP enzymes, particularly those within the CYP1–3 and CYP46 families, highlighting their functional diversity and the pathological consequences of their dysregulation on neurological health. It also examines the potential of cerebral CYP-based biomarkers to improve the diagnosis and treatment of neurodegenerative disorders, offering new avenues for therapeutic innovation.

## 1. Introduction

The cytochrome P450 (CYP or P450) superfamily comprises a remarkable assembly of heme-containing monooxygenase enzymes, renowned for their pivotal roles in catalyzing diverse oxidative metabolic pathways. These xenobiotic-metabolizing enzymes are integral to the biotransformation of molecules with physiological, pharmaceutical, and toxicological relevance, illuminating the complex biochemical pathways of life. Leveraging dioxygen along with reducing and proton equivalents, these tetrapyrrole heme–thiolate CYP enzymes exhibit unparalleled catalytic versatility, facilitating a wide range of reactions, including oxidative, peroxidative, and reductive transformations such as C–H bond hydroxylation, C=C bond epoxidation, heteroatom oxygenation, and numerous other uncommon reactions [1,2,3,4,5,6,7]. The functional diversity and substrate promiscuity of CYP enzymes make them indispensable across all domains of life, enabling the efficient metabolism of both endogenous (endobiotic) and exogenous (xenobiotic) compounds [8].

In the human genome, 57 genes encoding CYPs have been identified, systematically classified into 18 families and 44 subfamilies based on sequence homology [9,10,11,12,13,14]. Among these, the families CYP1–3 predominantly govern xenobiotic and physiological metabolism, whereas most of the other families are chiefly involved in the metabolism of endogenous substances [13,14,15,16]. The expression and function of human CYPs are intricately regulated by a multitude of genetic, physiological, pathophysiological, and environmental factors, serving as critical determinants of inter-individual variability in drug disposition and therapeutic outcomes. Perturbations in the expression and function of CYP enzymes can precipitate a spectrum of adverse effects, including drug–drug interactions, clinical non-response, atypical adverse reaction, and so on. It is crucial to acknowledge that cytochrome P450 reductase (CPR) plays an essential role in CYP monooxygenation by facilitating oxygen activation during the catalytic cycle [17,18]. Once regarded merely as an electron donor, CPR is now recognized as a functionality-determining factor or ‘regulating partner’, significantly influencing the efficiency and regulation of CYP-mediated reactions [10]. As one of the most expansive enzyme superfamilies, CYPs have garnered extensive research interest across a myriad of disciplines, including biochemistry, pharmacology, toxicology, genetics, and beyond [19,20,21,22,23]. In recent years, there has been a pronounced surge in the exploration of their pathophysiological implications, underscoring the multifaceted roles of these CYP enzymes in human health and disease [24,25,26,27,28,29,30].

**Figure 1:**
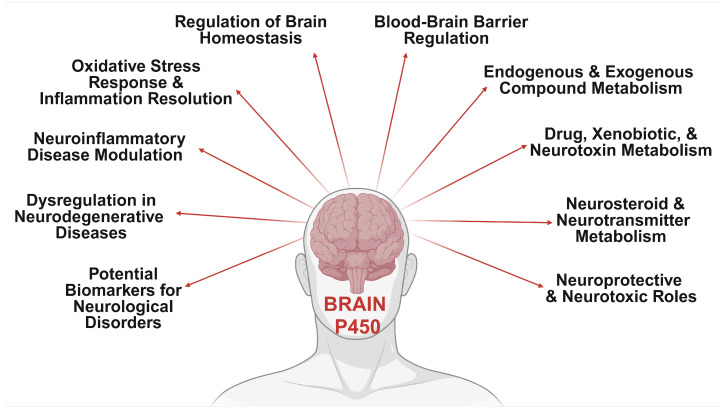
Diverse functions of brain cytochrome P450 enzymes. This diagram illustrates the multifaceted roles of brain-specific CYP enzymes, including their involvement in regulating brain homeostasis, blood–brain barrier function, and the metabolism of endogenous and exogenous compounds, including drugs and neurotoxins. Cerebral CYPs also play a critical role in neurosteroid and neurotransmitter metabolism, oxidative stress response, and modulating neuroinflammation, which are crucial for managing neurodegenerative diseases and neurological disorders. Additionally, CYP enzymes may serve as potential biomarkers for these diseases, influencing both neuroprotective and neurotoxic outcomes. These pathways underscore the essential role of cerebral CYP enzymes in maintaining brain homeostasis and adapting to physiological and pharmacological challenges. Generated using BioRender.

The promiscuous CYPs, historically associated with hepatic drug metabolism and detoxification, have recently been identified in the central nervous system (CNS), prompting intensive investigations into their intricate biochemical pathways within the brain [31,32,33,34,35,36,37,38]. While brain CYP levels are lower than in the liver, their impact on central substrate and metabolite concentrations is pivotal for modulating responses to these compounds. The heterogeneous distribution of CYPs in the brain, varying across regions and specific populations of neurons and glia, reveals insights into their functional significance and metabolic roles. Despite total cerebral CYP levels being substantially lower than hepatic levels, the localization of these enzymes to specific brain microenvironments allows for significant metabolic impacts within the CNS. Brain-specific CYP expression can be modulated by inducers, leading to organ-specific regulation and differential responses to psychoactive drugs and neurotoxins. This discovery challenges conventional perceptions and underscores the potential significance of these enzymes in local neuroactive substance metabolism, opening new avenues for understanding neurodegenerative, psychiatric, and neurological disorders. Dysregulation of brain CYP enzymes is increasingly recognized as a key contributor to disease progression, offering promising targets for therapeutic interventions. Recent studies have implicated brain-specific CYP isoforms in the metabolism of endogenous and exogenous neuroactive substances, neurotransmitters, neurosteroids, neurotoxins, and centrally acting compounds, contributing to xenobiotic metabolism within the CNS (Figure 1), challenging the notion of predominantly liver-based metabolism [34,35,36]. These findings establish a direct link between aberrant CYP activity and the development or exacerbation of several neurological disorders [31,32,37,39,40,41] such as Alzheimer’s disease (AD) [42,43,44], Parkinson’s disease (PD) [45,46], Huntington disease (HD) [47,48,49], schizophrenia [50,51,52], and other psychiatric and neurodegenerative conditions [53,54,55,56], unveiling intricate molecular pathophysiological mechanisms.

This review explores the emerging roles of CYP enzymes in the human brain, emphasizing their intricate localization, functional diversity, and the impact of their dysregulation on neurological health. The exploration commences with a detailed analysis of the neurological effects of cerebral CYP metabolism, highlighting their pivotal role in brain function and disease progression. The review also examines the differential expression of CYPs across brain regions and cell types, focusing on the major drug metabolizing enzymes of the CYP1, CYP2, CYP3, and CYP27 families, and the brain-specific CYP46A1, and their role in metabolizing endogenous and exogenous substances. In-depth discussions cover the implications of CYPs in neurodegenerative disorders (NDs), revealing the interplay between CYP activity and disease pathology. Additionally, it highlights the potential of CYP-based biomarkers for advancing diagnostic and therapeutic strategies in neurological disorders. By synthesizing recent findings, the review underscores the essential role of brain CYPs and paves the way for future research into their impact on drug metabolism, neurological health, and disease management.

## 2. Neurological Impacts of Brain CYP Metabolism

**Figure 2:**
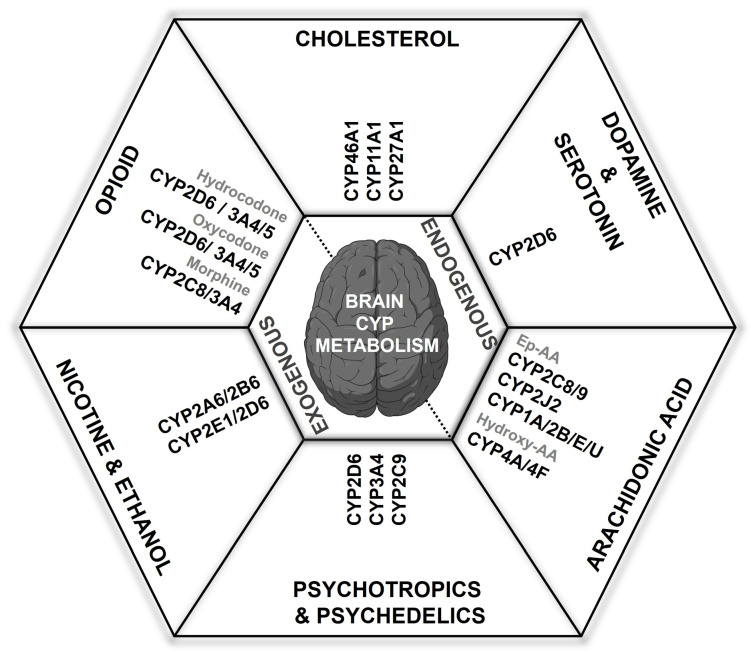
Schematic representation of cerebral CYP metabolism of endogenous and exogenous substances. The figure illustrates the major substrates metabolized by brain-specific CYP enzymes, organized into six key categories: cholesterol, dopamine and serotonin, arachidonic acid, psychotropics and psychedelics, nicotine and ethanol, and opioids. Each category highlights the relevant CYP isoforms (e.g., CYP46A1 for cholesterol, CYP2D6 for dopamine, serotonin, and opioids) and their corresponding substrates, showcasing the critical role of CYP enzymes in processing diverse compounds. This schematic underscores the specificity and versatility of CYP-mediated reactions essential for maintaining brain function and regulating pharmacological responses.

The human brain, as the second-largest lipid-rich organ, shows a significant connection between lipid metabolism, including fatty acids and cholesterol, and the regulation of brain energy balance, oxidative stress, and neuroinflammation [57]. The brain is susceptible to numerous toxic compounds which can cross the blood–brain barrier (BBB) to exert central effects. In the brain, CYP enzymes predominantly metabolize a variety of endogenous compounds such as sterols, fatty acids, hormones, vitamins, and eicosanoids [34,41,58]. Additionally, major cerebral CYP enzymes are implicated in the metabolism of diverse xenobiotics, including pharmaceuticals, psychoactive substances, and environmental toxins, into hydrophilic compounds, resulting in slower elimination from the brain [59]. This enzymatic activity substantially influences local xenobiotic metabolism, affecting the exposure and concentration of substrates and their metabolites within specific organs and tissues [34]. Understanding these mechanisms highlights the significance of cerebral CYP enzymes in neuroprotection and the metabolic processing of neurotoxic compounds (Figure 2).

The presence of cerebral CYPs facilitates the local metabolism of CNS-active drugs, impacting their activation and clearance within the brain. Moreover, cerebral CYPs might possess more potent local biotransformation capabilities compared to hepatic CYPs, potentially altering drug responses [35,60]. For example, CYP46A1, which metabolizes cholesterol, and CYP2D6, involved in dopamine and serotonin synthesis, are predominantly expressed in the brain, reflecting their critical physiological roles. These findings suggest that brain regions with low levels of drug-metabolizing CYP enzymes might influence treatment outcomes following clinical drug administration. Individual variability in the expression and activity of xenobiotic-metabolizing CYPs is primarily due to genetic polymorphisms and regulatory mechanisms. Furthermore, cerebral CYPs are inducible by xenobiotics, which can modulate their activity within the brain, contributing to variations in central xenobiotic metabolism and individualized responses to CNS-acting drugs and neurotoxins [37]. Although the relatively modest expression levels of these enzymes in the brain might not significantly impact systemic drug metabolism, localized cerebral CYPs in specific regions and cell types could profoundly affect local drug metabolism and overall brain function [39].

**Figure 3:**
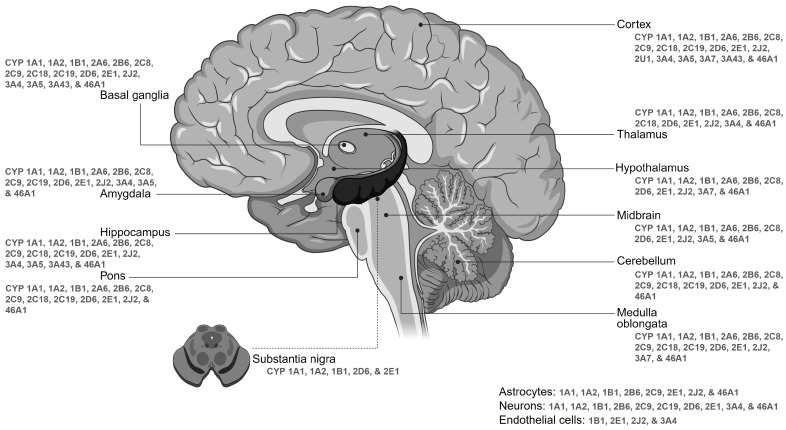
Regional localization and expression patterns of CYP enzymes in the human brain. This schematic illustrates the distribution of key drug-metabolizing CYP isoforms from the CYP1, CYP2, and CYP3 families, as well as the brain-specific CYP46A1, across various brain regions, based on data from the Human Protein Atlas [71], Genotype-Tissue Expression (GTEx) Project [78], Allen Brain Map [79], Gene Expression Omnibus (GEO) [80], EMBL-EBI Expression Atlas [81], and the corresponding literature [31,35,38,65,82,83]. Each brain region demonstrates a distinct CYP expression profile, highlighting the specialized roles these enzymes play in region-specific metabolic functions. Additionally, the figure depicts the localization of CYP enzymes across different cell types—astrocytes, neurons, and endothelial cells—emphasizing their cell-type-specific functions and contributions to local metabolism. This comprehensive view underscores the intricate spatial and cellular organization of cerebral CYP enzymes and their critical involvement in maintaining brain homeostasis, drug metabolism, and neuroprotection. Generated using BioRender.

Drug metabolism in the brain occurs at three primary sites: the blood–brain barrier, the blood–cerebrospinal fluid barrier, and within brain tissue [61,62,63,64]. This expanding field of research not only enhances our understanding of the brain function at the molecular level but also has significant implications for neurological disorders, psychiatric conditions, and therapeutic interventions. The brain’s ability to maintain homeostasis and defend against toxic compounds relies heavily on the proper functioning of CYPs in metabolizing endogenous substances [45]. Metabolic processes in the brain are vital, and factors beyond the BBB can influence pharmacological effects, rendering plasma levels unreliable for predicting drug reactions [32,45,65,66]. Understanding cerebral CYP metabolism is therefore crucial for comprehending the pharmacokinetics and impacts of CNS-acting drugs. The primary brain CYPs responsible for metabolizing most clinical drugs include CYP1A2, CYP3A4/5, CYP2C9, CYP2C19, CYP2D6, and CYP2E1. In this review, we prioritize the discussion of key human cerebral CYP families—specifically CYP1, CYP2, CYP3, and CYP46—due to their critical roles in cerebral metabolism and associated neurodegenerative diseases. The CYP1 family, including CYP1A1 and CYP1B1, is expressed across various brain regions and cell types (Figure 3), playing pivotal roles in metabolizing both endogenous and exogenous compounds and contributing significantly to neuroprotection. Their functions extend to neurotransmitter and neuroactive substance metabolism, impacting neurotransmission and neuronal signaling pathways, and influencing circadian rhythms and mood regulation [8,67,68]. Environmental factors and genetic variations can influence CYP1 enzyme levels, impacting neurochemical processing and vulnerability to neurotoxic damage. Increased CYP1A1 activity, observed in human brain glioma samples, generates reactive intermediates and DNA adducts, promoting carcinogenesis and indicating a potential link between brain CYP1A1 activity and susceptibility to environmental toxin-related brain cancers. The CYP2 family displays diverse expression patterns that are crucial to neurological processes, and significantly contribute to neuroprotection, neurotransmission, and overall brain function [8,69,70,71,72]. These enzymes are prominently expressed in neurons and glial cells (Figure 3). The distinct expression patterns of CYP2 enzymes across brain regions and cell types underscore their specialized roles in mediating neurochemical processes and responses to environmental stimuli. Isoforms of the CYP2 family, primarily CYP2D6, play essential roles in metabolizing endogenous compounds, synthesizing neurotransmitters like dopamine and serotonin, and detoxifying various exogenous substances. The CYP3 family, including CYP3A4 and CYP3A5, is expressed across various brain regions and cell types (Figure 3), contributing to the metabolism of endogenous compounds, hormones, and neurotransmitters, and the detoxification of exogenous substances [35,71,73,74,75]. This enzymatic activity promotes neuroprotection and influences neurological responses and pharmacological outcomes. The dynamic expression patterns of CYP3 enzymes across brain regions and developmental stages highlight their critical role in neurochemistry and brain health. Finally, the CYP46 family, particularly CYP46A1, plays a significant neurological role in cholesterol metabolism [58,76,77]. Dysregulation of cholesterol homeostasis can lead to various neurodegenerative conditions, emphasizing its critical role in brain health. The neuroprotective properties of hydroxy cholesterol metabolites contribute to synaptic plasticity, neurotransmitter release, and the prevention of cholesterol deposits that could contribute to neurodegeneration. A comprehensive understanding of cerebral CYP enzymes is essential for grasping brain metabolism, drug responses, and potential therapies for neurological disorders. Their expression in the human brain signifies an intricate interplay within the complex neurochemical environment. Notably, our understanding of drug metabolism in the CNS is currently limited, emphasizing the critical need for expanded research in this domain. Unraveling these mechanisms is pivotal for elucidating normal brain function and the pathogenesis of neurological disorders.

## 3. Differential Expression Patterns of Brain CYPs

The expression pattern of various CYP isoforms (Figure 3) across different brain regions highlights their crucial roles in synthesizing and degrading neuroactive substances, including neurotransmitters and neurosteroids. The initial assessment of CYP presence in the brain dates back to 1977, revealing the detection of 30 pmol/mg of CYP enzymes in the rat brain, constituting approximately 3% of the levels observed in the liver, with activity levels approximately 30 times lower [84]. Subsequent to this discovery, extensive endeavors have been undertaken to characterize various CYP enzymes, delineate their activity, and elucidate their expression profiles across different brain regions. Remarkably, the human brain harbors roughly 10 pmol/mg of the total CYP content, accounting for approximately 10% of the levels observed in liver microsomes. As of now, researchers have identified 41 out of the 57 known CYP enzymes within various brain regions [82,83]. Despite the lower expression levels of CYPs in the brain compared to the liver, they play significant roles in CNS substrate metabolism [39,63]. This differential expression leads to distinct concentration profiles of substrates and metabolites in the brain, potentially influencing the pharmacodynamics of drugs and the toxicity of neurotoxins. The distribution of CYP enzymes in the brain exhibits considerable variability across different regions of the CNS due to differences in cell types and densities [31,32,82,83]. This variability underscores the heterogeneous expression levels of CYP enzymes, suggesting significant implications within the brain (Table 1).

CYP subfamilies are present in various brain cell types, including diverse neuronal subtypes, astrocytes, glial cells, and endothelial cells, with localization within cell bodies, processes, mitochondria, and plasma membrane fractions of neurons [31]. In the CNS, astrocytes and microglia are pivotal cell populations that respond to neuronal injuries [85]. These glial cells can undergo morphological changes, alter the expression of neurotrophic and neurotoxic factors, and influence their interactions in response to various forms of neurological damage, including those caused by toxicants, through a process known as gliosis. Beyond their roles in providing nutritional and structural support to neurons, astrocytes are essential for controlling neurotoxins within the CNS. Astrocytes express significantly higher levels of CYP enzymes, approximately 2.7 times greater than neurons, indicating distinct functional roles between astrocytic and neuronal CYP systems [85]. This high expression level underscores the importance of astrocytes as the first line of defense against xenobiotics. Astrocyte-specific CYP-dependent monooxygenases play a crucial role in establishing the brain as the target organ for various toxic agents.

In the human brain, CYP enzymes are distributed extensively across neurons and glial cells, spanning from cell bodies to dendrites, axons, synaptic terminals, and various glial components. Interestingly, some neurons exhibit CYP expression levels comparable to or higher than hepatocytes, emphasizing the organizational and functional significance within brain cells [35,37,65,82]. This widespread distribution of CYP enzymes is especially significant in crucial brain regions, including the cortex, hippocampus, cerebellum, basal ganglia, medulla oblongata, pons, and substantia nigra, where they are abundantly enriched (Figure 3) [82]. Remarkably, the cerebellum and brainstem exhibit the highest concentrations of CYP enzymes, whereas lower levels are detected in areas such as the hippocampus, microvasculature, and striatum [35,86,87,88]. The expression patterns of certain xenobiotic-metabolizing enzymes are consistently observed throughout the mammalian brain [39]. Overall, the distribution of CYP mRNA, protein, and enzymatic activity shows a diffuse subcellular pattern across various brain regions in humans [31,32,33,34,37], with key enzymes like CYP1A, 2B, 2C, 2D, 2E, 3A, and 46A1 primarily localized in neurons and astrocytes, while others are predominately expressed in both glial and neuron cells, exhibiting notable cellular specificity (Figure 3) (Table 1) [65,82,83,89].

Studies using mRNA quantification have identified isoforms of CYP1, CYP2, and CYP46 as major enzymes, accounting for over 90% of mRNA levels and exhibiting selective distribution across different brain regions [82,83]. Notably, CYP1A1 demonstrates widespread expression in the human brain, particularly in neurons such as pyramidal neurons in the cerebral cortex and hippocampus, purkinje and granule neurons in the cerebellum, and midbrain reticular neurons [89]. Similarly, CYP2D6 is notably abundant in specific neuronal and glial cell types, including pyramidal neurons, granular cells, purkinje cells, and glial cells, across diverse brain regions predominantly in the cerebellum, the cortex, the amygdaloid complex, the olfactory bulbs, as well as in the hippocampus, the substantia nigra, and the striatum [65,90]. Studies suggest that although the expression of CYP2D6 enzyme levels increases with age, PD patients demonstrate a 40% reduction in its expression compared to those with a healthy brain [91], potentially compromising their ability to deactivate neurotoxins associated with pathology. Additionally, CYP2E1 is predominantly found in neurons, notably enriched in pyramidal neurons of the frontal cortex, hippocampus, and cerebellum [69]. Moreover, CYP3A4 may be present in both epithelial and neuronal cells within the brain [31]. Furthermore, CYP46A1 exhibits selective expressions in astrocytes and neurons in specific brain areas such as the hippocampus, cortex, cerebellum, dentate gyrus, and thalamus [37,71,77,92,93]. Alongside the brain-specific CYP46, isoforms of CYP7 and CYP26 are also predominantly expressed in the brain according to the Human Protein Atlas Database [71]. The expression of CYP2B in the brain varies among species, with humans showing low constitutive levels, particularly within hippocampal neurons and astrocytes near blood vessels in the frontal cortex [39]. However, elevated levels of CYP2B6 are observed in specific brain regions of individuals with a history of smoking and alcohol use disorder, suggesting the inducibility of CYP2B6 in the human brain. On the other hand, CYP2C, 2D, and 3A show broad expression across key regions of the cerebral cortex, hippocampus, amygdala, basal ganglia, and cerebellum, with additional detection in areas such as the olfactory bulbs, hypothalamus, thalamus, and brainstem [75,89,94].

The exploration of CYP expression and activity in the brain encompasses a range of sophisticated methodologies, including mRNA analysis in specific brain regions or the entire brain (Table 1) [34]. Despite pronounced inter-individual variability, a multitude of CYP isoforms have been ascertained within the human brain, with proteomic methodologies serving as pivotal tools in elucidating their spatial distribution across distinct brain regions. Notably, CYP proteins are infrequently identified in comprehensive proteomic analyses of brain tissues, a phenomenon potentially attributable to their comparatively diminished expression levels in contrast to hepatic tissues, alongside technical challenges associated with the detection of CYP peptides. Beyond mRNA analysis, quantification of CYP protein levels in the brain is achieved through Western blotting, while diverse labeling techniques like in situ and in cyto methods are utilized to map the spatial distribution of CYP proteins across various brain regions, tissues, and cells. Despite challenges in assessing CYP metabolic activity due to low protein levels and rapid brain tissue degradation postmortem, in vitro evaluation becomes feasible by analyzing enzymatic product formation following incubation with CYP-specific substrates. Conversely, in vivo assessment involves analyzing substrate and CYP-derived metabolite concentrations in brain tissue or dialysate post-substrate administration, providing crucial insights into in vivo substrate metabolism within the CNS [90,95].

Notwithstanding substantial progress, numerous studies investigating brain-specific CYP isoforms have yielded conflicting findings, exposing inconsistencies in the expression of these enzymes within the brain. Particularly, contradictions have been noted in the key DMEs such as CYP1, 2D, and 3A isoforms concerning their documented expression profiles [35,65,96,97,98], including those in the Human Protein Atlas database [71]. Likewise, differences between mRNA and protein data may arise as a consequence of differential effects of post-transcriptional regulation on protein levels relative to mRNA levels [37]. While mRNA molecules are more susceptible to degradation than proteins, the specificity and potential for cross-reactivity of antibodies utilized in investigations targeting CYP enzymes can exhibit variability, primarily stemming from their typical generation against hepatic CYP isoforms that may diverge from those prevalent in the CNS. These discrepancies underscore the necessity for clear expression profile studies, as current mappings of cerebral CYP expression based on existing literature and databases may contradict native in vivo expression profiles (Figure 3) (Table 1). The inconsistencies appear to stem from various factors, including sex- and species-specific variations; racial or individual diversity; differences in sample sources or preparations, storage conditions, environmental influences, and analytical methodologies; and the high degree of homology among CYP isoforms. Conflicting perspectives on the cellular and regional localization of CYP expression in the brain highlight the complexities in characterizing CYP expression within specific brain regions. Nevertheless, the existence of CYPs in particular brain areas suggests a potential role for cerebral CYP metabolism within those regions. This potential role warrants broader investigation, especially considering the key findings that imply a direct connection between cerebral CYP metabolism and several NDs (elaborated on in later sections). Future research should focus on more precise methodologies, including advanced single-cell transcriptomics, proteomics, and spatial metabolomics, to map the expression and activity of CYP enzymes in different brain regions and cell types. Additionally, experimental approaches using human brain tissue, organoids, or in vivo models, combined with CRISPR or other gene-editing techniques, could provide deeper insights into the functional significance of CYPs in neuroprotection, drug metabolism, and the pathogenesis of NDs. This broader investigation will contribute to a more comprehensive understanding of cerebral CYP metabolism and its role in neurological health and disease.

**Table 1:** Comprehensive profile of cerebral CYP isoform characteristics. This table summarizes the key expression and functional attributes of major CYP isoforms as detected in various brain regions. It presents detailed data on gene expression, protein levels, transcript levels, and enzyme activity, along with information on the experimental models and analytical techniques used to generate these findings. The data have been compiled from multiple sources, including the Human Protein Atlas [71], and other established datasets like the Genotype-Tissue Expression (GTEx) Project [78], Allen Brain Map [79], Gene Expression Omnibus (GEO) [80], and EMBL-EBI Expression Atlas [81]. The selected isoforms are highlighted based on their documented roles in xenobiotic metabolism, cholesterol homeostasis, and neurodegenerative processes, as well as their distinct expression patterns across different brain regions.

CYP Family	Isoform	Brain Regions	Gene Expression	Protein Levels	Transcript Levels	Enzyme Activity	Experimental Models	Techniques Employed	References
CYP1	CYP1A1	Cortex, Hippocampus, Cerebellum	High in cerebral cortex and cerebellum	Predominantly in neurons	Elevated levels noted	Detectable activity in neuronal cultures	Animal and human studies	qPCR, Western blot, immunohistochemistry	[99,100,101]
	CYP1B1	Cortex, Hippocampus, Other Regions	Present in neural tissue; varies	Significant in blood–brain barrier, human neurons, and astrocytes	Variable across samples	Active in xenobiotic and steroid metabolism	Genetic models	IHC, in situ hybridization	[102,103,104,105,106]
CYP2	CYP2D6	Cortex, Cerebellum, Hippocampus, Amygdala, Olfactory Bulbs	High in substantia nigra	Significant across brain regions (hippocampus, thalamus, hypothalamus, cortex)	Variability in expression observed	Critical for drug metabolism	Transgenic and knockout models	qPCR, LC-MS, Western blot	[97,107,108,109,110]
	CYP2C19	Cortex, Hippocampus	Expressed variably across regions	Detectable levels notably in pyramidal neurons	High variability in expression	Modulated by pharmacological agents	Experimental models	PCR, IHC, drug response assays	[107,111,112,113,114,115]
CYP3	CYP3A4	Frontal Cortex, Hippocampus	Expressed in hippocampus and cortex	Neuronal expression high in certain areas	High variability reported	Important for drug metabolism	Animal models	Immunohistochemistry	[63,116,117]
	CYP3A5	Cortex, Hippocampus	Expressed variably across brain areas	Levels vary across brain regions	High in specific regions	Documented activity in drug metabolism	Human and animal studies	Western blot, ELISA	[31,116,117,118]
CYP27	CYP27A1	Cortex, Hippocampus, Cerebellum, Microglia	Elevated in brain regions involved in cholesterol metabolism	Variable across studies, notably high	Confirmed RNA levels across regions	Active in cholesterol oxidation	Knockout and transgenic studies	RT-PCR, Western blot	[119,120,121,122,123]
CYP46	CYP46A1	Cortex, Hippocampus, Cerebellum	Primarily in neurons	Expression linked to neurodegeneration	High in specific regions	Essential for cholesterol metabolism	Gene therapy models	qPCR, immunofluorescence	[39,92,124,125,126]

## 4. Metabolic and Regulatory Interactions of Brain CYPs

### 4.1. Endogenous Substance Dynamics of Cerebral CYPs 

CYP enzymes in the brain play a crucial role in the intricate synthesis and metabolism of various endogenous substances, including neurotransmitters such as dopamine and serotonin, and essential fatty acids like arachidonic acid and cholesterol (Figure 2). The interaction between cerebral CYP enzymes and the BBB is vital for regulating the levels of these substances within the CNS. This dynamic regulation is essential for maintaining CNS homeostasis and modulating pharmacological responses, highlighting the significance of cerebral CYPs in ensuring optimal brain function and health.

#### 4.1.1. Cerebral CYPs in Cholesterol Metabolism

Cholesterol, a steroid alcohol derived from acetyl coenzyme A, serves as an essential structural component of cell plasma membranes, significantly influencing their fluidity, rigidity, curvature, and overall architecture. The brain is particularly rich in cholesterol, which is crucial not only for maintaining eukaryotic membrane integrity but also as a precursor for various neurosteroids, oxysterols, bile acids, vitamin D, and steroid hormones. Although the brain represents only 2.1% of total body mass, it contains roughly 23% of the body’s cholesterol [77,127]. This cholesterol exists predominantly in an unesterified state; approximately 70% is localized in the myelin sheaths of oligodendrocytes, while the remainder is distributed among cell membranes. In human gray and white matter, cholesterol constitutes 22% and 27.5% of the dry weight, respectively, influencing the membrane ordering, fluidity, permeability, and functionality of membrane-bound proteins and lipid rafts [77,126]. Consequently, cholesterol is vital for processes such as electrical impulse transmission along axons, synaptogenesis, and synaptic function, and is essential for brain development, axonal myelination, and lipid raft formation [38,40,128,129,130]. Cholesterol biosynthesis in the brain is intricate and tightly regulated, involving over 30 enzymes and requiring substantial energy and oxygen [131]. In the mammalian CNS, cholesterol synthesis proceeds through multiple steps via two primary pathways—the Bloch pathway, likely active in astrocytes, and the Kandutsch–Russell pathway, presumably occurring in neurons [126,132]. The synthesized cholesterol is then delivered to neurons to support the formation of axons, dendrites, synapses, and synaptic vesicles. Notably, the brain contains about 25% of the body’s cholesterol, underscoring its critical role in neurological functions. Due to the blood–brain barrier (BBB), cholesterol metabolism in the brain differs from that in peripheral tissues; most CNS cholesterol is synthesized de novo by glial cells, particularly astrocytes, because it cannot freely diffuse across the BBB [77,126,133,134]. Thus, the majority of cerebral cholesterol is produced locally.

**Figure 4:**
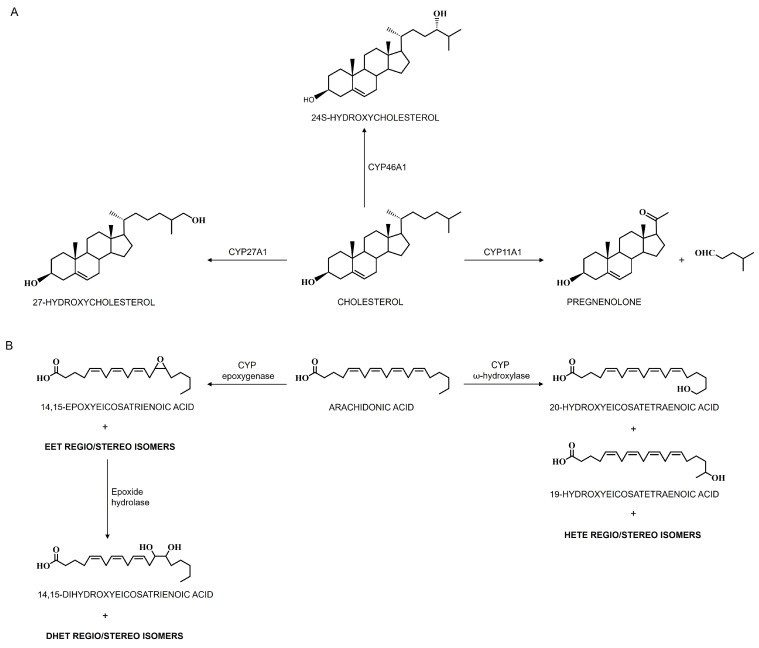
Cerebral CYP enzymes in major endogenous substance metabolism. (**A**) Cholesterol metabolism: Overview of key steps in the conversion of cholesterol and its derivatives mediated by cerebral CYP enzymes, highlighting pathways involved in neurosteroid synthesis and oxysterol production. Cholesterol is converted into pregnenolone by CYP11A1, while CYP46A1 catalyzes the production of 24S-hydroxycholesterol, and CYP27A1 converts cholesterol into 27-hydroxycholesterol. (**B**) Polyunsaturated fatty acid metabolism: Representation of major pathways catalyzed by cerebral CYP enzymes in the metabolism of polyunsaturated fatty acids (PUFAs). CYP epoxygenase converts arachidonic acid into 14,15-epoxyeicosatrienoic acid, which is further metabolized into 14,15-dihydroxyeicosatrienoic acid by epoxide hydrolase. Additionally, CYP ω-hydroxylase facilitates the formation of hydroxylated derivatives, such as 20-hydroxyeicosatetraenoic acid and 19-hydroxyeicosatetraenoic acid.

Cholesterol homeostasis in the brain is maintained through a balance of biosynthesis and elimination. A key elimination mechanism is cholesterol hydroxylation by CYP enzymes: CNS-specific CYP46A1 converts cholesterol to 24S-hydroxycholesterol (24-HC), mitochondrial CYP27A1 produces 27-hydroxycholesterol, and steroidogenic CYP11A1 generates pregnenolone [77,126]. Notably, the primary route involves CYP46A1—accounting for 75–85% of cholesterol elimination in the human brain (Figure 4A) [56,77,126,133], with in vitro reconstituted systems demonstrating its efficient conversion of cholesterol to 24-HC and, to a lesser degree, to 24,25- and 24,27-dihydroxycholesterols [58,92]. Unlike cholesterol, 24-HC rapidly diffuses out of cells, crossing the BBB to enter systemic circulation (~98%) or cerebrospinal fluid (CSF) (~2%), and is subsequently delivered to the liver for further degradation into bile acids [91]. High CYP46A1 expression renders serum 24-HC a reliable biomarker of brain enzyme activity that reflects the brain-to-liver volume ratio [77,126]. By facilitating cholesterol efflux and activating cholesterol synthesis pathways, CYP46A1 ensures continuous brain cholesterol turnover, its activity potentially driving a 20% daily turnover in specific neuronal populations, with CYP46A1 responsible for 99% of brain 24-HC, which comprises 60–80% of serum 24-HC. Modulating CYP46A1 activity alters brain 24-HC levels, thereby influencing key receptors: liver X receptors (LXRs), which regulate cholesterol elimination, fatty acid and triglyceride biosynthesis, glucose metabolism, and immune–inflammatory responses, and N-methyl-D-aspartate receptors (NMDARs), for which 24-HC acts as a positive allosteric modulator critical for excitatory neurotransmission, synaptic plasticity, and learning [77,126]. Beyond cholesterol maintenance, CYP46A1 activity also impacts essential cellular pathways including gene transcription, endocytosis, misfolded protein clearance, vesicular transport, and synaptic transmission.

Intriguingly, changes in CYP expression and function can profoundly affect cholesterol metabolism, contributing to the pathophysiology of neurodegenerative disorders. Elevated levels of cholesterol metabolites in the CSF are associated with increased brain cholesterol in postmortem AD brains, which exhibit senile plaques and neurofibrillary tangles [135]. Cholesterol plays several critical roles in CNS cells; its rigid, apolar ring structure enables deep integration within membranes, reducing membrane fluidity and dynamics. Elevated cholesterol in membranes impedes the lateral diffusion of lipids and proteins, adversely affecting the conformation, stability, and binding properties of membrane proteins, including neurotransmitter receptors. Disruptions in cholesterol homeostasis, often linked to altered cerebral CYP46A1 activity, have been implicated in the development of NDs [40,47]. Although multiple studies suggest that imbalanced brain cholesterol regulation is associated with various neurological, inflammatory, and vascular disorders across different life stages, the precise mechanisms underlying these disturbances remain incompletely understood. Maintaining cholesterol equilibrium in the brain is essential for processes such as synaptic plasticity and cognitive functions related to learning and memory [42,43,131]. 

The interplay between steroid hormones, cholesterol metabolism, and CYP enzymes in the brain underscores their critical role in neuroprotection and their potential involvement in NDs [42,48,136]. Notably, CYP46A1 offers a promising therapeutic avenue for neurological conditions, as evidenced by alterations in 24S-hydroxycholesterol—a marker closely linked to its function—across disorders such as AD, PD, HD, Niemann–Pick disease type C, and spinocerebellar ataxia [44,49,53,56,137,138]. Interestingly, pharmacological activation of CYP46A1 has also shown promise in reducing the progression of colorectal cancer and inhibiting glioblastoma, an aggressive brain tumor, with several underlying mechanisms involving improvements in neurotransmission, vesicular and endosomal trafficking, proteasome and autophagy functions, reduced neuronal atrophy, and decreased microgliosis [139,140]. Conversely, a deficiency in CYP46A1 impairs memory and learning, likely due to reduced long-term potentiation resulting from decreased protein prenylation. In addition, region-specific reductions in CYP46A1 expression are associated with the development of NDs characterized by neuronal degeneration, lysosomal dysfunction, abnormal amyloid precursor protein recruitment, endoplasmic reticulum stress, and abnormal tau phosphorylation [42]. Given these associations, preclinical investigations have focused on therapeutic strategies targeting CYP46A1 activity, with the modulation of CYP activity or expression levels holding promise for mitigating neurotoxicity, enhancing neuroprotective mechanisms, and improving treatment efficacy in these debilitating conditions. Nonetheless, further breakthroughs are imperative to elucidate the precise mechanisms and fully realize the therapeutic potential of targeting cerebral CYPs in NDs.

#### 4.1.2. Cerebral CYPs in Dopamine Metabolism

Dopamine, a crucial neurotransmitter for brain communication, is integral to various physiological and behavioral functions such as mood regulation, cognition, reward processing, motivation, movement control, and emotional regulation [41]. Dysregulation of dopamine levels or receptors is associated with psychiatric conditions like depression, schizophrenia, and addiction. Excessive dopamine activity in certain brain regions can lead to psychosis, while reduced signaling is linked to depressive symptoms. Neurodegenerative conditions like Parkinson’s disease, characterized by the loss of dopamine-producing neurons, result in motor impairments due to disrupted dopamine function [32].

**Figure 5:**
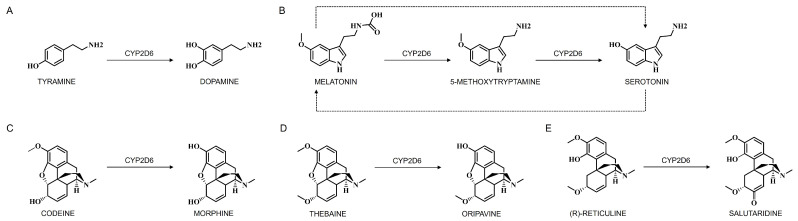
Selected biotransformation reactions mediated by CYP2D6 in the brain. This figure illustrates key metabolic reactions catalyzed by CYP2D6 in the central nervous system: (**A**) Hydroxylation of tyramine: CYP2D6 catalyzes the conversion of tyramine to dopamine, a neurotransmitter crucial for motor control and reward mechanisms. (**B**) O-demethylation of 5-methoxytryptamine and deacetylation of melatonin: CYP2D6 is involved in the metabolism of serotonin and melatonin, converting melatonin into 5-methoxytryptamine, which can undergo further metabolism to regenerate serotonin, underscoring the complex interplay between these pathways. (**C**) 3-O-demethylation of codeine: CYP2D6 mediates the conversion of codeine into its active metabolite, morphine, responsible for its analgesic effects. (**D**) 3-O-demethylation of thebaine: Thebaine is metabolized into oripavine by CYP2D6, an intermediate step in the biosynthesis of certain opioids. (**E**) Phenol coupling of (R)-reticuline: CYP2D6 facilitates the conversion of (R)-reticuline into salutaridine, a key neuroactive benzylisoquinoline alkaloid in the biosynthesis of morphinan compounds. These reactions demonstrate the multifunctional role of CYP2D6 in maintaining neurochemical homeostasis and its involvement in the metabolism of both therapeutic drugs and endogenous compounds.

A significant pathway of dopamine metabolism involves cerebral CYP enzymes, particularly human CYP2D6, which hydroxylates tyramine to synthesize dopamine (Figure 5A) under specific conditions, such as when classical synthesis pathways are compromised [141,142]. The presence of tyramine and CYP2D activity in catecholaminergic brain structures suggests that despite low CYP2D levels and the inability of dietary tyramine to cross the BBB, dopamine levels remain elevated due to the active classical biosynthetic pathway from tyrosine [143]. In the conventional synthesis pathway, phenylalanine is converted to tyrosine by phenylalanine hydroxylase, which is then oxidized by tyrosine hydroxylase to form L-dopa. L-dopa is further metabolized into dopamine by DOPA decarboxylase [143]. Dopamine is then processed into norepinephrine by dopamine β-hydroxylase and into epinephrine by phenylethanolamine N-methyltransferase. Intriguingly, tyramine can also be synthesized in the brain, particularly in the basal ganglia, through aromatic hydroxylation of phenylethylamine or tyrosine decarboxylation, providing a substrate for CYP2D in dopamine synthesis. The presence of tyramine and CYP2D activity in catecholaminergic brain structures suggests the role of CYP2D in dopamine synthesis [141,142]. Interestingly, brain microsomes with CYP2D isoforms can convert tyramine to dopamine, with m-tyramine being a preferred substrate over p-tyramine. Although p-tyramine is generally found in higher concentrations in the brain, the more efficient hydroxylation of m-tyramine in vitro might not translate to a significant difference in vivo due to the higher concentration of p-tyramine.

While CYP2D4 is considered the primary CYP2D isoform in the rat brain, which has six isoforms (CYP2D1–5 and CYP2D18), humans have only one representative isoform, CYP2D6, with all CYP2D isoforms sharing a high degree of overall amino acid sequence identity, ranging from 71% to 99%. Human CYP2D6 is more efficient than rat CYP2D isoforms (2D2/4/18) at catalyzing this reaction, indicating its potential importance under specific conditions, such as the inhibition of classical pathways or induction of human brain CYP2D6 [143,144,145]. These findings suggest that CYP2D-mediated dopamine biosynthesis might be more significant in humans than in rodents, assuming similar in vivo kinetic parameters and enzyme expression levels between the species. The reaction was inhibited by the CYP2D inhibitor quinine and by anti-CYP2D4 antibodies, suggesting that CYP2D isoforms govern the tyramine hydroxylation to dopamine in the brain [141,142]. An alternative dopamine biosynthesis pathway mediated by cerebral CYP2D isoforms may provide an additional route to tyrosine hydroxylase-mediated dopamine synthesis. While dopamine metabolism is well established, unexplored metabolites likely exist across various brain regions. Other CYP enzymes, such as CYP2E1, may also modulate dopamine levels, particularly in areas like the substantia nigra, where CYP2E1 inhibition has been linked to increased extracellular dopamine and altered L-dopa metabolite patterns [32]. This complexity enhances our understanding of neurotransmitter regulation in the brain and underscores the intricate biochemical processes essential for neurotransmitter homeostasis and function.

#### 4.1.3. Cerebral CYPs in Serotonin Metabolism

Serotonin serves as a neurotransmitter within the CNS, facilitating communication between neurons and regulating neural functions. It is primarily sourced from dietary intake, originating from the amino acid L-tryptophan, and is transported into the brain through specific amino acid carriers. Upon release by midbrain neurons, serotonin plays essential roles in a wide range of physiological and behavioral functions within the body. Its involvement in psychiatric disorders such as depression, anxiety, schizophrenia, and obsessive–compulsive disorder underscores its significance in regulating fundamental behaviors and emotional responses [146]. Furthermore, serotonin is integral to the mechanism of action of certain psychotropic medications like serotonin reuptake inhibitors.

The enzymatic pathway responsible for serotonin synthesis from tryptophan involves sequential steps, starting with tryptophan hydroxylase converting tryptophan into 5-hydroxytryptophan (5-HTP), which is then decarboxylated by 5-hydroxytryptophan decarboxylase to produce serotonin (5-HT) [147,148]. An interesting facet of serotonin synthesis is the alternative pathway mediated by CYP2D6, which converts 5-methoxytryptamine (5-MT) directly into serotonin (5-HT) through O-demethylation (Figure 5B). The sources of 5-MT include melatonin deacetylation, serotonin O-methylation, or 5-methoxytryptophan decarboxylation in brain and liver tissues. Notably, these compounds share an indole nucleus derived from tryptophan, facilitating efficient recycling in their biosynthetic pathways. This conversion of 5-HT from 5-MT via CYP2D indicates a cycle that preserves the biological activities of serotonin and other indoleamines by delaying their elimination, maintaining a dynamic cycle [147,148]. While tryptophan hydroxylase 2 remains the rate-limiting enzyme in serotonin production, the CYP2D6-catalyzed conversion of 5-MT to serotonin has been observed in vitro with human and rat cDNA-expressed CYP2D isoforms, as well as in liver and brain tissues [147,148,149].

Research on human CYP2D6-transgenic mice has shown increased levels of 5-HT and 5-hydroxyindole acetic acid in the brain, emphasizing the role of brain CYP2D6 in catalyzing serotonin formation from 5-MT [107]. Additionally, experiments with quinine-induced decreased activity of cerebellar CYP2D demonstrated behavioral deficits and increased anxiety-related behavior, highlighting the importance of brain CYP2D in serotonin (5-HT) formation [150]. Moreover, melatonin supports 5-HT synthesis via CYP2D in the brain through deacetylation to 5-MT (Figure 5B). The involvement of CYP2D6 in the regeneration of serotonin from 5-methoxytryptamine highlights its potential impact on neurophysiological and pathophysiological mechanisms in both vertebrate and invertebrate nervous systems. Elevated brain CYP2D levels during depressive disorders may lead to increased melatonin consumption, contributing to an efficient serotonin–melatonin–serotonin cycle that influences sleep disorders in patients with depressive disorders [149,151]. In vivo, melatonin facilitates the CYP2D-mediated synthesis of serotonin from 5-MT in the brain, thereby completing the serotonin–melatonin–serotonin biochemical cycle. The conversion of exogenous melatonin to the neurotransmitter serotonin represents a newly identified aspect of its pharmacological activity.

#### 4.1.4. Cerebral CYPs in Polyunsaturated Fatty Acid Metabolism

The role of polyunsaturated fatty acids (PUFAs) in neural functions is significant due to their abundance in neural tissues, with arachidonic acid (AA) being the most prevalent omega-6 PUFA in brain tissue lipid content, primarily esterified to choline glycerophospholipids on cell plasma membranes at a ratio of 10,000:1 (esterified/unesterified) [152,153,154]. The brain exhibits robust de novo synthesis of epoxyeicosapentaenoic acids (Ep-PUFAs) from parent PUFAs via CYP enzymes, with subsequent rapid degradation of these Ep-PUFAs by epoxide hydrolases (EHs) like sEH in neuronal cells [155]. While the CYP pathway seems to be the predominant route for Ep-PUFA production in the brain, cellular uptake may also transport Ep-PUFAs to the brain. The primary elimination mechanism for Ep-PUFAs involves EH-mediated hydrolysis to more polar 1,2-diols, with potential processes such as spontaneous hydration, beta-oxidation, chain elongation, and reincorporation into phospholipids maintaining Ep-PUFA homeostasis [156].

The main CYP enzymes responsible for metabolizing arachidonic acid (AA) to either epoxyeicosatrienoic acids (EETs) via epoxygenase activity or to hydroxyeicosatetraenoic acids (HETEs) via ω-hydroxylase activity (Figure 4B) include members of the CYP2 family—specifically, CYP2B, CYP2C, and CYP2J—for AA epoxygenase, and isoforms from the CYP1 and CYP4 subfamilies for AA hydroxylation [152,157]. CYP epoxygenases introduce an oxygen atom across AA’s double bonds to produce four EET isomers (5,6-, 8,9-, 11,12-, and 14,15-EET), with the primary human enzymes being CYP2C8/9 and CYP2J2, although CYP1A, CYP2B, and CYP2E also exhibit epoxygenase activity to some extent [158]. The formation of EETs is isoform-specific, yielding either R,S- or S,R-stereoisomers depending on the double bond involved. Conversely, CYP hydroxylases add hydroxyl groups to AA to form various HETE derivatives—including 5-, 8-, 9-, 11-, 12-, 15-, 16-, 17-, 18-, 19-, and 20-HETE—primarily via the CYP4A and CYP4F subfamilies, which hydroxylate AA at the terminal methyl group to produce 20-HETE as the major product and 19-HETE as a minor product [152,153]. Both the CYP epoxygenase and hydroxylase families exhibit regio- and stereoselectivity; the specific positions and orientations of oxygen insertion determine the biological activities of these metabolites, influencing critical functions such as vascular regulation, inflammation, and neurotransmission. Notably, all EETs and HETEs, except for 20-HETE, can exist as either R- or S-enantiomers. Moreover, the same CYP isoform may shift its preference between epoxygenase and hydroxylase activity when the substrate changes from AA to linoleic acid (LA). Additionally, the membrane-bound epoxide hydrolase (EH) enzyme hydrolyzes xenobiotic epoxides and Ep-PUFAs into dihydroxyeicosatrienoic acids (DHETs) (Figure 4B), which are elevated in AD patients, thereby further highlighting the significant role of CYP enzymes in both physiological and pathological processes [152,153].

Brain oxylipin levels are markedly influenced by dietary lipids. In mouse brains, omega-3 PUFA supplementation increases CYP-derived metabolites from EPA and DHA while reducing those from AA, and enhances pro-resolving oxylipin synthesis in the hippocampus, offering neurodevelopmental benefits and mitigating neurodegenerative processes [159,160,161]. Conversely, in rats, an omega-6-rich diet raises AA and LA oxylipins and lowers EPA-derived oxylipins in the cerebral cortex, whereas an LA-deficient diet reduces LPS-induced PGE2 formation [162,163]. Notably, a high DHA-to-AA ratio does not necessarily elevate DHA-derived oxylipins. Additionally, AA is metabolized into anandamide—which is further processed by CYP3A4, CYP4F2, and CYP2D6—and both anandamide and its metabolites exhibit anti-inflammatory properties [164]. Furthermore, CYP1B1 and CYP2U1, which convert AA to HETEs, are present in human brain microvasculature, and their expression in astrocytes is modulated by glutamate-induced neuron–astrocyte signaling via the mGlu5 receptor, all influencing CYP-mediated AA metabolism [165].

Under pathophysiological conditions, certain CYP enzymes can impact various physiological processes such as inflammation, brain development, blood flow regulation, infant growth and immune response by modulating AA metabolism [166]. EETs, released from astrocytes, neurons, and endothelial cells, act as potent vasodilators to improve blood flow or as anti-inflammatory agents to safeguard against cerebrovascular diseases [167]. Inflammation significantly reduces the expression and activity of CYP epoxygenases not only in the brain but also in other organs like the heart, kidneys, and liver. This suppression leads to decreased EET formation, intensifying the inflammatory response and creating a cycle of exacerbation. Studies have demonstrated that inhibiting endogenous EET production with the selective CYP epoxygenase inhibitor N-(methylsulfonyl)-2-(2-propynyloxy)-benzenehexanamide (MS-PPOH) exacerbates Aβ-induced mitochondrial dysfunction in astrocytes [168]. Conversely, pretreatment of primary hippocampal astrocyte cultures with exogenous 11,12-EET or 14,15-EET prevents mitochondrial depolarization and fragmentation caused by Aβ. Targeting CYP epoxygenases could be a therapeutic approach for chronic cerebrovascular diseases and brain inflammatory disorders. Additionally, EETs play a neuroprotective role by mitigating oxidative stress and neuroinflammation, suggesting their potential in treating neurological conditions such as Parkinson’s disease [168,169,170].

### 4.2. Exogenous Substance Dynamics of Cerebral CYPs

Cerebral CYP enzymes are vital for metabolizing and interacting with various exogenous substances that enter the CNS, including drugs, environmental toxins, opioids, nicotine, psychedelics, psychotic substances, and other foreign compounds (Figure 2). This function is essential for drug metabolism, detoxification, and responding to environmental exposures within CNS. Interestingly, the response variability to centrally acting drugs often does not align with circulating drug concentrations, suggesting complex factors beyond systemic bioavailability. A significant contributor to this variability is the unique CYP-mediated drug metabolism in the brain, resulting in distinct drug and metabolite levels between central and peripheral compartments. For instance, CYP2D6, which is highly expressed in the brain, is known to influence the metabolism of drugs like antidepressants (e.g., fluoxetine) [171] and antipsychotics (e.g., risperidone) [172], contributing to patient-specific variability in therapeutic outcomes. To target brain CYPs without affecting liver function, strategies involve selective induction and inhibition approaches. While some inducers increase CYP expression in both hepatic and extrahepatic tissues, others can specifically upregulate CYP expression in the brain [101]. A key method to achieve brain-specific CYP modulation involves administering low doses of pharmacological inhibitors directly into the brain, thus avoiding systemic liver effects. Irreversible inhibitors are often used to selectively deactivate specific brain CYPs, with a carefully timed interval between pretreatment and substrate administration, allowing for accurate behavioral assessments without affecting liver CYP activity [33,173,174,175,176]. Understanding how cerebral CYPs handle exogenous substances is critical for understanding drug effectiveness, toxicity, and potential therapeutic interventions for neurological disorders and environmental neurotoxicity.

#### 4.2.1. Cerebral CYPs in Opioid Metabolism

Opioids are CNS depressants and act upon opioid receptors (μ, κ, and δ) that are found in the brain, spinal cord, and periphery. Most commonly, opioids have a higher affinity for the μ-opioid receptor [177]. Most opioids are metabolized by CYP enzymes, primarily by CYP3A4 and CYP2D6 [178,179,180]. Hydrocodone, oxycodone, and morphine are among the most widely prescribed or used opioid analgesics [177]. Opioid analgesics are widely used in clinical practice for a wide variety of pain management plans for both chronic and acute pain. Opioids are the classical pain treatment for patients who suffer from moderate to severe pain. Opioids are often prescribed in combination with multiple other drugs, especially in patient populations who typically are prescribed a large drug regimen. Not only do opioids have narrow therapeutic indexes and are extensively used, but they have the potential to cause severe toxicity.

##### Hydrocodone Metabolism by Cerebral CYPs

Hydrocodone is commonly used for pain management, and its association with the human brain is a critical aspect of understanding its effects, risks, and therapeutic use. Hydrocodone acts primarily on opioid receptors in the CNS, including μ-opioid receptors (MORs) [181,182]. Activation of MORs leads to analgesia, euphoria, and respiratory depression, contributing to its potential for misuse and addiction [183,184]. Hydrocodone and its metabolites also modulate neurotransmitter systems in the brain, such as dopamine and serotonin, contributing to its analgesic and psychoactive effects. The interplay between hydrocodone metabolism and brain effects has clinical implications for opioid therapy. Chronic hydrocodone use can result in neuroadaptive changes in the brain, such as downregulation of opioid receptors and increased sensitivity to pain [181,182].

**Figure 6:**
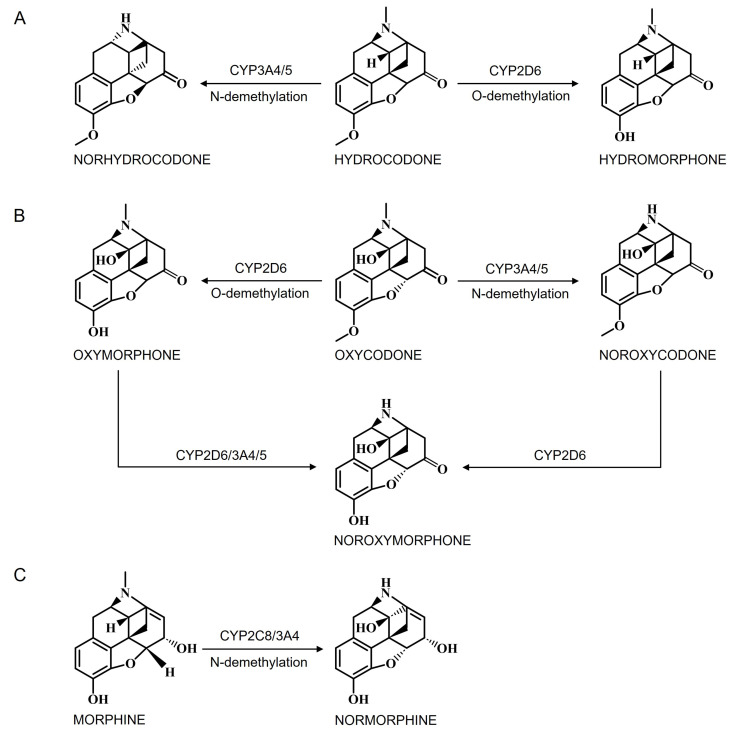
Cerebral CYP enzymes in major exogenous substance metabolism—opioid biotransformation. (**A**) Hydrocodone metabolism: CYP3A4/5 catalyzes N-demethylation of hydrocodone to norhydrocodone, while CYP2D6 mediates O-demethylation to form the active metabolite hydromorphone. (**B**) Oxycodone metabolism: Oxycodone undergoes O-demethylation by CYP2D6 to produce the active metabolite oxymorphone and N-demethylation by CYP3A4/5 to form noroxycodone. Noroxycodone and oxymorphone can be further metabolized to noroxymorphone through CYP2D6 and CYP3A4/5. (**C**) Morphine metabolism: Morphine is converted to normorphine via N-demethylation mediated by CYP2C8 and CYP3A4. These pathways emphasize the significant role of cerebral CYP enzymes in the metabolism, pharmacokinetics, and pharmacodynamics of opioid drugs within the central nervous system.

Hydrocodone metabolism by cerebral CYP enzymes is a crucial aspect of understanding the pharmacokinetics and pharmacodynamics of opioid medication. Greater than 50% of the total hydrocodone dose is metabolized by CYP-mediated metabolism [177]. Hydrocodone undergoes extensive metabolism primarily by CYP2D6 (Figure 6A), which converts hydrocodone via O-demethylation to its active metabolite, hydromorphone, which possesses higher affinity and activity at opioid receptors compared to hydrocodone itself [185]. The activity of CYP2D6 can vary widely among individuals due to genetic polymorphisms. Some individuals are rapid metabolizers with increased enzyme activity, leading to more rapid conversion of hydrocodone to hydromorphone and potentially enhanced analgesic effects [180,186]. Conversely, poor metabolizers have reduced enzyme activity, resulting in slower metabolism of hydrocodone and potentially reduced efficacy [187]. In addition to CYP2D6, CYP3A4/5 also contributes to the formation of norhydrocodone, a less active metabolite of hydrocodone, through N-demethylation (Figure 6A) [172]. As CYP3A4 and CYP3A5 are highly homologous and metabolize the same substrates, the contribution of CYP3A5 to hydrocodone metabolism is still unclear [74]. Although CYP3A4 plays a minor role compared to CYP2D6, it contributes to the overall metabolism and elimination of hydrocodone from the body.

The metabolism of hydrocodone can be influenced by drug interactions that affect CYP enzyme activity. Inhibitors of CYP2D6, such as certain antidepressants (e.g., fluoxetine, paroxetine), can decrease the conversion of hydrocodone to hydromorphone, potentially reducing its efficacy. On the other hand, inducers of CYP2D6, such as rifampicin, may increase hydrocodone metabolism and lead to decreased opioid effects [187]. Understanding the role of CYP enzymes in hydrocodone metabolism is important in clinical practice. Healthcare providers need to consider genetic variability in CYP2D6 activity and individual differences in metabolism, receptor sensitivity, and potential CNS effects when prescribing hydrocodone to optimize pain management and minimize adverse outcomes. Monitoring drug interactions that affect CYP metabolism is also essential to optimize therapeutic outcomes and minimize adverse effects. Understanding these metabolic and neural mechanisms is crucial for safe and effective opioid use in clinical practice.

##### Oxycodone Metabolism by Cerebral CYPs

Oxycodone is also a potent opioid analgesic like hydrocodone and has significant implications for the brain due to its pharmacological effects and potential for abuse and addiction. When oxycodone enters the bloodstream, it can readily cross the blood–brain barrier, gaining access to the CNS and exerting its effects on various brain regions. The metabolism of oxycodone is similar to that of hydrocodone. The primary oxidative pathway for oxycodone is by N-demethylation by CYP3A4/5 to form noroxycodone (Figure 6B), an inactive metabolite [188]. CYP3A5 is active in oxycodone metabolism, which is consistent with many previous studies demonstrating that most CYP3A4 substrates may also be metabolized by CYP3A5 due to their high sequence homology [189]. However, the actual contribution of CYP3A5 (vs. CYP3A4) to oxycodone metabolism has not been firmly established [189,190]. In addition to N-demethylation, oxycodone undergoes O-demethylation via CYP2D6 to produce oxymorphone (Figure 6B), an active metabolite with strong analgesic properties [191]. Noroxycodone, however, has weak antinociceptive effects and is considered inactive compared to oxymorphone. Interestingly, both oxymorphone and noroxycodone are further metabolized to noroxymorphone by CYP3A4/5 and CYP2D6, respectively (Figure 6B). Oxycodone itself exhibits analgesic effects, and inhibition of CYP2D6 does not diminish its antinociceptive properties or opioid-related side effects, suggesting that oxycodone’s activity is not solely dependent on its conversion to oxymorphone [188,189]. Furthermore, oxycodone crosses the BBB effectively and is approximately 1.5 times more potent than morphine when administered orally (WHO, 2018), though both drugs are categorized as medium-potency opioids [192]. Notably, oxymorphone, oxycodone’s active metabolite, has a significantly higher affinity for the μ-opioid receptor, approximately 40 times greater than oxycodone, and is 10 times more potent than oxycodone when administered intravenously [177,192,193]. These differences in metabolism and receptor affinity highlight the complexity of oxycodone’s pharmacokinetics and pharmacodynamics.

##### Morphine Metabolism by Cerebral CYPs

The presence of brain CYP enzymes, particularly CYP2D6 and CYP3A4, indicates a potential local metabolism of morphine, another opioid analgesic widely used for pain management in the CNS [177,194]. Morphine is N-demethylated by CYP3A4 and CYP2C8 to form a minor metabolite, normorphine (Figure 6C), which can be further metabolized to two glucuronide metabolites [195].

Intriguingly, CYP2D6 catalyzes three reactions in the biosynthesis of morphine: it catalyzes the 3-O-demethylation of codeine to morphine (Figure 5C), the 3-O-demethylation of thebaine to oripavine (Figure 5D), and the phenol coupling of (R)-reticuline to salutaridine (Figure 5E) [196]. The CYP3A4 and CYP2D6 isoforms are key players in the metabolism of morphine, leading to the formation of metabolites like morphine-3-glucuronide (M3G) and morphine-6-glucuronide (M6G) through glucuronidation processes [177,195,197]. CYP3A4 predominantly catalyzes the conversion of morphine to M3G, while CYP2D6 plays a significant role in the formation of M6G. M6G is renowned for its potent analgesic properties and contributes significantly to morphine’s overall pharmacological effects. Although M6G is pharmacologically active, M3G is considered pharmacologically inactive but may contribute to certain adverse effects associated with morphine use, such as neuroexcitatory effects and opioid-induced hyperalgesia [197]. M6G is considered to be active and may partially contribute to morphine’s analgesic effect. Studies have shown that the potency of M6G is equal to or more active than morphine depending on the route of administration [198]. However, several studies suggest that the contribution of M6G to morphine’s analgesic effects is likely small due to it accounting for only 10% of circulating metabolite in plasma and because it does not easily cross the BBB as compared to morphine [198,199]. However, some studies suggest that M6G may have a major role in morphine analgesia, although the analgesic efficacy was found to not be dependent on M6G plasma concentration [177]. In contrast, M3G does not exhibit analgesic effects and was shown to antagonize the analgesic effects of both morphine and M6G [200]. Studies have shown that M3G causes neuroexcitatory effects such as hyperalgesia, allodynia, and myoclonus.

Factors influencing the metabolism of morphine by CYP enzymes include genetic variations, drug interactions with inhibitors or inducers of these enzymes, and physiological conditions like liver impairment [197]. Behavioral characteristics like anxiety and impulsivity have been related to CYP2D6 polymorphisms and their relationship with serotonin/dopamine balance, drug–drug interaction, efficacy, and adverse effects in psychotic disorders treatments [201,202]. Understanding the intricate metabolism of morphine by cerebral CYP enzymes is crucial for optimizing its therapeutic efficacy while minimizing potential adverse effects, thereby ensuring safe and effective pain management strategies. The exact significance and extent of morphine metabolism by brain CYP enzymes are still being investigated. However, studies have suggested that local metabolism in the brain may contribute to the pharmacological effects and potential side effects of morphine, including its analgesic properties and neuroexcitatory effects [195].

##### Cannabinoid Metabolism by Cerebral CYPs

Cerebral CYP enzymes, in addition to metabolizing opioids, play a crucial role in the metabolism of cannabinoids, compounds that interact with cannabinoid receptors within the endocannabinoid system [203]. While cannabinoids are primarily metabolized by hepatic CYP enzymes, brain-expressed CYPs, including CYP1A1/2, CYP2C9, CYP2C19, CYP2D6, and CYP3A4, also contribute significantly to their metabolism within the CNS [204,205]. This metabolic activity influences the bioavailability, therapeutic efficacy, and elimination of cannabinoids, directly impacting their effects on cognition, pain, neuroinflammation, and neuroprotection.

The two most well-characterized cannabinoids, Δ9-tetrahydrocannabinol (THC) and cannabidiol (CBD), undergo extensive metabolism mediated by CYP enzymes [206,207]. THC, the psychoactive component of cannabis, is predominantly metabolized by CYP2C9 and CYP3A4. CYP2C9 converts THC to 11-hydroxy-THC (11-OH-THC), a potent psychoactive metabolite capable of crossing the BBB and exerting significant CNS effects such as euphoria, analgesia, and neuroprotection [208]. This metabolite is subsequently oxidized to 11-nor-9-carboxy-THC (THC-COOH), an inactive form that is excreted. Variations in THC metabolism are influenced by genetic polymorphisms in CYP2C9, leading to inter-individual differences in the duration and intensity of THC’s psychoactive and therapeutic effects [209]. CBD, a non-psychoactive cannabinoid, is metabolized by CYP3A4 and CYP2C19 into its major active metabolite, 7-hydroxy-CBD (7-OH-CBD) [210]. This metabolite exhibits notable anti-inflammatory and neuroprotective properties. CYP3A4 further metabolizes 7-OH-CBD into inactive compounds, which are eventually eliminated [208]. The metabolism of CBD is also subject to genetic variability, particularly in CYP2C19, where polymorphisms may result in altered CBD metabolism, affecting its therapeutic potential and the risk of side effects [208]. In patients with impaired CYP2C19 activity, CBD may accumulate, potentially enhancing its anti-inflammatory and neuroprotective effects but also increasing the likelihood of adverse reactions.

The co-administration of cannabinoids with opioids, especially in chronic pain management, presents a valuable therapeutic strategy, as cannabinoids may enhance analgesia while reducing the required dose of opioids [211]. This “opioid-sparing effect” can mitigate opioid-related side effects such as respiratory depression, dependency, and overdose [212]. However, the effectiveness of this approach is influenced by CYP enzyme polymorphisms, which affect the metabolism of both cannabinoids and opioids [213]. Genetic variations in CYP genes can lead to altered drug metabolism rates, resulting in variability in drug responses, particularly in patients with NDs, where the need to maintain cognitive function and neuroprotection is critical [208].

In NDs such as AD, PD, and multiple sclerosis, cannabinoids are increasingly being explored for their neuroprotective, anti-inflammatory, and antioxidant effects [214]. The metabolic activity of CYP enzymes modulates the therapeutic potential of cannabinoids in these conditions. Cannabinoids can reduce neuroinflammation and oxidative stress, both of which are key drivers of neurodegeneration. For instance, the production of 7-OH-CBD, which is dependent on CYP3A4 and CYP2C19 activity, plays a pivotal role in mediating CBD’s anti-inflammatory effects [215]. Genetic variability in these enzymes could alter the efficacy of CBD in reducing neuroinflammation, potentially impacting the progression of neurodegenerative diseases.

Furthermore, the modulation of cannabinoid receptors, particularly CB1, may protect neurons from excitotoxicity, a common pathological feature in neurodegenerative diseases characterized by excessive glutamate release [216]. CYP enzymes, by controlling the metabolism and clearance of cannabinoids, influence the ability of these compounds to protect neurons from glutamate-induced damage. Thus, the interplay between CYP-mediated metabolism and cannabinoid efficacy is crucial for developing effective therapeutic strategies for neuroprotection [217]. One of the most significant factors affecting cannabinoid metabolism is the presence of genetic polymorphisms in CYP enzymes [209]. Variants in CYP2C9, CYP3A4, and CYP2C19 can lead to marked differences in the metabolism of THC and CBD, influencing their therapeutic effects and safety profiles [208,218]. For instance, the CYP2C9*3 variant slows THC metabolism, prolonging psychoactive effects and increasing both therapeutic benefits and risks. Similarly, CYP2C19 polymorphisms can modify CBD metabolism, potentially enhancing its neuroprotective effects but also increasing the risk of sedation.

Given the variability in CYP-mediated cannabinoid metabolism, personalized medicine approaches are essential for optimizing cannabinoid therapy and cannabinoid-associated coagulopathy, particularly in the context of neurodegenerative diseases. Understanding an individual’s CYP genetic profile could improve dosing strategies and enhance therapeutic outcomes while minimizing adverse effects. Future research should focus on identifying specific CYP polymorphisms that influence cannabinoid metabolism in neurodegenerative patients, paving the way for more tailored and effective treatment regimens that harness the full neuroprotective potential of cannabinoids.

#### 4.2.2. Cerebral CYPs in Nicotine and Ethanol Metabolism

**Figure 7:**
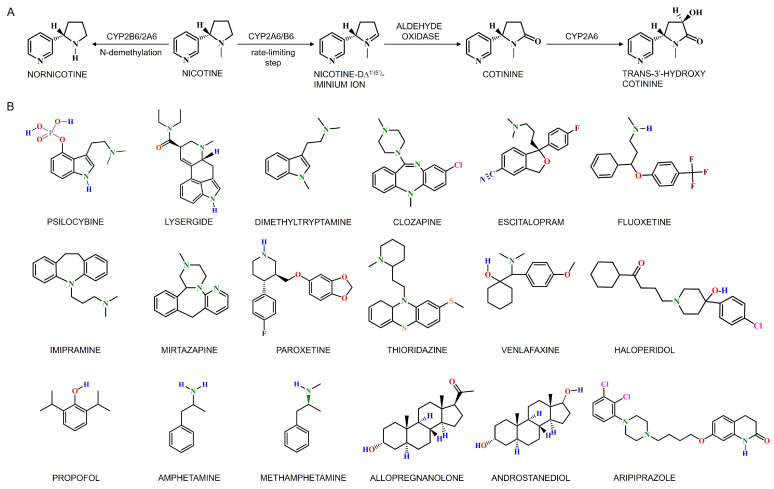
Cerebral CYP enzymes in major exogenous substance metabolism—nicotine and psychoactive compound metabolism. (**A**) Nicotine metabolism: Depiction of the metabolic pathway of nicotine, including its N-demethylation to nornicotine by CYP2B6/2A6, formation of the iminium ion intermediate by CYP2A6/B6 (rate-limiting step), oxidation by aldehyde oxidase to cotinine, and hydroxylation by CYP2A6 to trans-3′-hydroxycotinine. These steps highlight CYP enzymes’ role in nicotine clearance and regulation of its activity in the brain. (**B**) Metabolism of psychoactive and psychedelic substances: Structures of representative compounds such as psilocybin, lysergide, dimethyltryptamine, and various psychotropic drugs (e.g., fluoxetine, clozapine, amphetamine). Key chemical modifications mediated by CYP enzymes, such as hydroxylation (marked in blue), oxidation (marked in red), and other functional group transformations, are highlighted to demonstrate the diversity of CYP-mediated reactions. This figure emphasizes the broad scope of cerebral CYP enzymes in processing nicotine and a wide range of psychoactive drugs, shaping their pharmacological effects and clearance profiles.

Nicotine, the primary psychoactive compound in tobacco, modulates dopamine activity in the midbrain, influencing smokers to maintain specific nicotine levels within defined thresholds in the brain [219]. It is the primary component of cigarette smoke that induces reward from smoking and withdrawal during smoking cessation. The duration of nicotine’s action in the CNS is largely influenced by its metabolism and clearance rate. Although nicotine is primarily metabolized in the liver, extrahepatic enzymes also play a role in biotransformation. Cerebral CYP enzymes are involved in the deactivation of nicotine and the activation of carcinogenic compounds in tobacco [220]. The CYP2 family, especially CYP2A6, is primarily responsible for nicotine metabolism in humans and other species [221]. The rate at which nicotine is inactivated and cleared depends mainly on the activity of CYP2A6. Cotinine is the main metabolite of nicotine, with 70 to 80% of nicotine being converted to cotinine; CYP2A6 is responsible for approximately 90% of this metabolism [221]. This metabolic conversion occurs in two steps: first, nicotine is metabolized to the nicotine-D∆1′(5′)-iminium ion by CYP2A6 (the rate-limiting step), and then this intermediate metabolite is converted to cotinine by the cytosolic enzyme aldehyde oxidase (Figure 7A) [219,222]. Cotinine is further metabolized to trans-3′-hydroxycotinine (3HC) entirely by the CYP2A6 enzyme. Briefly, the primary nicotine metabolic pathway involves its inactivation to cotinine, followed by cotinine’s further metabolism to 3HC, processes which are 90% and 100% mediated by CYP2A6, respectively [219,222]. The long half-life of cotinine (COT) and the dependency of 3HC formation on cotinine result in stable relative levels of these metabolites over time in regular smokers. The ratio of these primary metabolites, 3HC/COT (the nicotine metabolite ratio, NMR), serves as a phenotypic biomarker of CYP2A6 enzymatic activity and the rate of nicotine metabolism [223]. The rate of nicotine metabolism, and thus the NMR, varies significantly among individuals and is associated with different smoking behaviors.

The nicotine metabolite ratio (NMR) is strongly correlated with the overall rate of nicotine clearance, as CYP2A6 mediates 70–80% of nicotine metabolism—accounting for 95% of its total clearance compared to 5% via renal excretion [223]. Supporting its use as a biomarker, individuals with the CYP2A6*4/4 genotype (a complete gene deletion) do not produce 3HC, indicating a lack of cotinine conversion in these CYP2A6-null subjects [223]. Among regular smokers, nicotine dependence is linked to CYP2A6 genotype and metabolism rate, with reduced-function alleles or slower CYP2A6 activity (lower NMR quartiles) associated with lower nicotine dependence and fewer cigarettes per day [219,224,225]. A meta-analysis of 18 studies across diverse populations confirmed that smokers with one or more reduced-function CYP2A6 alleles smoke significantly fewer cigarettes than normal metabolizers (CYP2A61/*1) [226]. Variations in nicotine metabolism also influence cessation outcomes; slower metabolizers are more likely to quit spontaneously and show higher quit rates with nicotine replacement therapy (NRT) compared to faster metabolizers, who may benefit more from bupropion or varenicline—given that bupropion is metabolized by CYP2B6 [219,227]. These findings underscore the critical role of genetic variability in nicotine metabolism and its impact on smoking behavior and cessation success [225,227,228,229].

Intriguingly, nicotine exposure upregulates the cerebral expression of CYP2B6, CYP2D6, and CYP2E1, illustrating the broader role of the CYP2 family in nicotine metabolism. In particular, CYP2B6 appears crucial in brain nicotine metabolism and addictive behaviors, as individuals with compromised CYP2B6 function exhibit heightened cravings and increased relapse during smoking cessation [222]. Human CYP2B6 shares significant amino acid similarity and substrate affinity with its rat homolog CYP2B1, highlighting its versatile capacity to metabolize nicotine and other psychoactive substances [230,231]. The regional distribution of CYP2B expression in the brain underscores spatial heterogeneity in enzyme activity, with nicotine specifically inducing CYP2B activity in the brain, independent of hepatic pathways. Nicotine is metabolized via N-demethylation to form nornicotine—a process mediated by both CYP2B6 and CYP2A6 (Figure 7A)—and while this pathway is minor in systemic clearance, some studies suggest that nornicotine may be a major brain metabolite capable of crossing the BBB [232,233]. Moreover, smokers exhibit higher cerebral CYP2B6 protein levels than non-smokers, and approximately 10% of nicotine metabolism to cotinine is mediated by CYP2B6 [234]. Additionally, heavy smokers display elevated brain CYP2D6 levels, particularly in the basal ganglia, although nicotine’s influence on hepatic CYP2D6 and CYP2B6 remains minimal, preserving overall metabolic stability [225,232,233,234].

Furthermore, CYP2A6, along with CYP2A7 and CYP2A13, is involved in activating and metabolizing local carcinogens in the human lungs [204]. Notably, smokers exhibit lower nicotine clearance than non-smokers, possibly due to downregulation of hepatic CYP2A6. In the context of nicotine–dopamine metabolism, smoking status correlates more strongly with gene–gene interactions [e.g., between CYP2A6 and Catechol-O-Methyltransferase [229], dopamine D2 receptor TaqIB [235], monoamine oxidase A [236], and the nicotine acetylcholine receptor gene [237]] than with individual genes alone. Although CYP regulation in hepatic and certain non-hepatic tissues is well documented, little is known about its regulation in the brain. Moreover, specific cerebral CYPs—particularly CYP2B6 and CYP2E1—are inducible by both ethanol and nicotine, with the patterns of induction varying by organ and species (humans, African green monkeys, and rats) [238,239]. Ethanol-induced upregulation of brain CYP2E1 has been associated with increased reactive oxygen species (ROS) levels and decreased synaptic protein expression, partly due to peroxisome proliferator-activated receptor alpha downregulation, thereby contributing to neurotoxic effects [70,240]. Additionally, cerebral CYP2D6 significantly metabolizes addictive substances like methamphetamine, influencing their pharmacological responses when its activity is heightened [241,242]. Elevated levels of these CYP enzymes in smokers, particularly in specific brain cell populations, may confer neuroprotective benefits by neutralizing neurotoxins—a hypothesis supported by the reduced risk of Parkinson’s disease observed in smokers compared to non-smokers, suggesting a potential neuroprotective function of CYP2D6 [32,243,244].

Nicotine has been shown to protect against dopaminergic neuron degeneration in experimental settings, suggesting a neuroprotective role that may also reduce inflammatory responses in the nervous system [245,246,247]. Although elevated CYP enzyme levels—whether due to genetic factors or nicotine-induced upregulation—are linked to neural oxidative stress, they might simultaneously mitigate PD progression by enhancing the detoxification of neurotoxic compounds. Proposed mechanisms include activation of nicotinic acetylcholine receptors, which increases dopamine release, and inhibition of brain monoamine oxidases by tobacco smoke, thereby reducing the formation of PD-inducing toxins such as MPTP [245,246,247]. Earlier investigations have highlighted the involvement of cerebral CYPs in nicotine metabolism and their potential link to PD; however, these studies are limited by preliminary data, inadequate control groups, and unidentified genetic variants. Despite recent studies establishing a clear association between nicotine exposure and PD [245,246,247,248,249,250], the precise impact of cerebral CYP-mediated nicotine metabolism on PD remains unclear. This gap underscores the critical need for comprehensive research into human cerebral CYPs, their role in nicotine metabolism, and their potential relevance to PD (elaborated on in Section 5.2.1).

#### 4.2.3. Cerebral CYPs in Psychotropic and Psychedelic Metabolism

Recent studies have underscored the critical role of cerebral CYP enzymes in the metabolism of various psychotropic medications and psychedelics within the CNS. These interactions can lead to significant drug–drug interactions, as substances that induce or inhibit CYP activity can alter the metabolism and effects of these drugs [33]. Psychotropic medications, including antidepressants, antipsychotics, and anxiolytics, undergo metabolism by cerebral CYP enzymes, affecting their pharmacokinetics and pharmacodynamics [41,127,251]. Similarly, psychedelics such as psilocybin, LSD, and DMT are metabolized by brain CYP enzymes (Figure 7B), which influence their duration and intensity of effects [59,252,253]. Chronic mild stress and the use of antidepressants can enhance the local brain metabolism of CYP2D substrates, such as neurosteroids, neurotransmitters, and psychotropics [33,41]. Intriguingly, these drugs can influence CYP enzymes through various mechanisms involving the nervous, hormonal, and immune systems, as well as the liver [127]. The impact of these drugs on CYP enzymes in the brain often differs from their effects in the liver and can vary by region. Because psychotropic drugs affect CYP activity in both the liver and the brain, they can modify their pharmacological effects at both pharmacokinetic and pharmacodynamic levels. While liver CYP enzymes are responsible for the biotransformation of psychotropic drugs, brain CYP enzymes are crucial for the local metabolism of these drugs and endogenous neuroactive substances, such as neurosteroids [41,127,251]. Additionally, brain CYP enzymes are involved in alternative pathways of neurotransmitter biosynthesis, including those for dopamine and serotonin as discussed earlier.

Many drugs employed in psychiatric treatment undergo metabolism by diverse cerebral xenobiotic-metabolizing enzymes, notably the CYP2C and CYP2D subfamily isoforms, which are expressed in the brain [59,235,236,237,238,239,240,241,242,243,244,245,246,247,248,249,250,251,252,253,254,255]. For instance, the conversion of psilocybin to its active form, psilocin, is partly mediated by cerebral CYP enzymes [255]. Antidepressants (Figure 7B) like clozapine, escitalopram, fluoxetine, imipramine, mirtazapine, paroxetine, thioridazine, and venlafaxine exhibit varying effects on CYP2D activity, with some increasing CYP2D activity in specific brain regions while decreasing it in the liver [33,41,151]. Additionally, CYP2D is pivotal in opioid metabolism as discussed earlier. In post-operative scenarios, higher oxycodone doses are often required for effective pain relief, particularly in smokers, likely due to increased CYP2D6 activity, resulting in accelerated drug metabolism and reduced brain drug levels, impacting analgesic effectiveness [173,175,256,257].

CYP2D6 plays a pivotal role in metabolizing various antipsychotic drugs (Figure 7B), including both first- and second-generation ones like haloperidol, known for its tendency to trigger extrapyramidal side effects [258]. In the brain, CYP2D activity appears to counteract the long-term effects of haloperidol, while its inhibition leads to increased drug levels and worsened side effects. The cerebral metabolism of haloperidol by CYP2D6 significantly impacts the severity of these side effects over prolonged periods. Furthermore, cerebral CYP2D activity may influence acute catalepsy response, potentially through the creation of neurotoxic metabolites. Notably, nicotine treatment reduces haloperidol-induced movements, possibly by stimulating CYP2D. Smoking habits correlate with reduced chronic side effects, suggesting CYP2D6’s involvement in antipsychotic metabolism in the brain [34,91]. Interestingly, the variability in CYP2B6 expression in the human brain potentially influences brain propofol levels, affecting anesthesia depth, supported by rodent CYP2B counterparts [259].

Additionally, changes in brain CYP2D activity, as evidenced by its involvement in metabolizing psychostimulants (Figure 7B) like amphetamine and methamphetamine, can independently impact central and behavioral responses to both acute and chronic drug administration, regardless of variations in liver metabolism or peripheral drug concentrations [34,241]. Individuals with genetically reduced CYP2D6 activity display increased sensitivity to methamphetamine dose adjustments, affecting subjective responses such as stimulation, euphoria, and perceived drug effects, even in the absence of discernible differences in peripheral drug levels [34]. This suggests that variations in brain CYP2D6 activity may influence central drug concentrations and subsequent responses. Among methamphetamine users, higher levels of nicotine dependence correlate with increased methamphetamine consumption, consistent with the idea that individuals with elevated brain CYP2D6 activity, often found in smokers, metabolize methamphetamine more rapidly, requiring greater intake to achieve desired central effects [34,241,260]. CYP3A4 and CYP2D6 also regulate the plasma concentrations of aripiprazole, an antipsychotic used in treating schizophrenia, and affect the symptoms and cognitive function of patients receiving risperidone [261,262]. Furthermore, CYP2D6 is implicated in the metabolism of neuroactive steroids (e.g., allopregnanolone and androstanediol) (Figure 7B), which has implications for conditions like schizophrenia and bipolar disorder.

Apparently, the CNS plays a role in the central neuroendocrine and neuroimmune regulation of liver CYP enzymes, suggesting a complex bidirectional relationship [34,241,263]. This interaction underscores the necessity of investigating the effects of neuroactive drugs on CYP activity in vivo to fully understand the mechanisms of drug–enzyme interactions, including neuroendocrine and neuroimmune modulation. Personalized medicine approaches that account for individual genetic profiles and CYP enzyme activity can optimize treatment outcomes and minimize adverse effects. Understanding the role of cerebral CYP enzymes in metabolizing psychedelics and psychotropic medications enhances our knowledge of how these substances affect brain function and behavior, informing safer and more effective therapeutic use.

## 5. Brain CYPs: Implications in Neurodegenerative Diseases

CYP-mediated reactions in the brain can produce neurotoxic reactive oxygen species (ROS), which disrupt the balance between the neuroprotective and neurotoxic roles of specific cerebral CYP enzymes. These enzymes are essential for CNS function as they synthesize and degrade various neuroactive compounds, neurotransmitters, and hormones, thereby influencing neuronal communication. However, neuroinflammation, triggered by processes such as oxidative damage, microglial and astrocyte activation, and the release of inflammatory molecules like cytokines and chemokines, can alter the expression and activity of cerebral CYP enzymes [35]. While inflammation generally reduces CYP activity [264], certain cerebral CYPs may be upregulated during neuroinflammatory states [265], leading to altered drug metabolism and clinical outcomes. This modulation may contribute to the progression of neurodegenerative and neuropsychiatric disorders, as well as traumatic brain injury.

**Figure 8:**
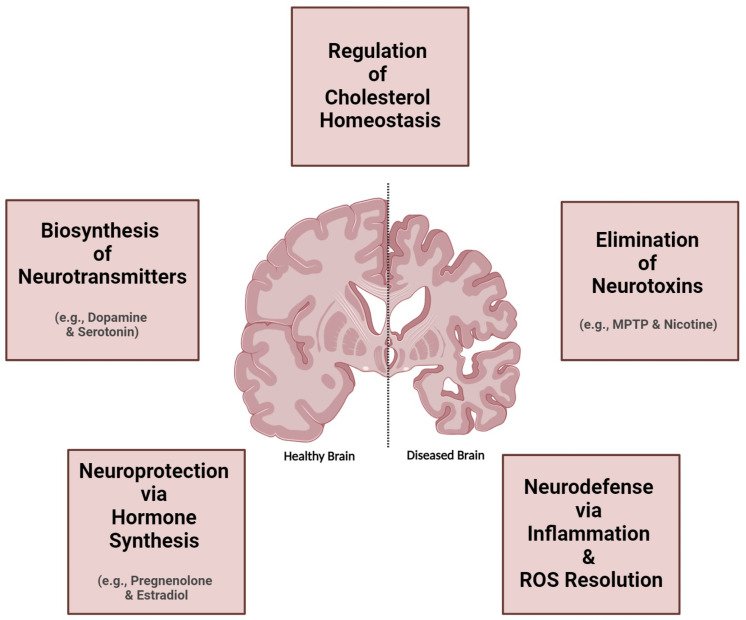
Defense mechanisms of cerebral CYP enzymes against brain diseases. The figure illustrates several vital processes that contribute to the maintenance of brain health (**left** side) and their potential dysregulation in diseased states (**right** side). These processes collectively contribute to brain homeostasis, with dysfunction in these CYP-mediated mechanisms potentially contributing to the development of neurodegenerative disorders. Generated using BioRender.

Cerebral CYP enzymes are particularly important for metabolizing endogenous substances such as dopamine and ROS, as well as for clearing amyloid-beta peptides, underscoring their critical role in the progression of neurodegenerative diseases. Notably, genetic variations in these enzymes can also influence drug metabolism and treatment responses, making them key factors in therapeutic interventions for NDs. Although evidence directly linking neuroinflammation to changes in cerebral CYP activity is still limited, further research into these mechanisms may provide insights into targeted therapies for neurological conditions (Figure 8).

### 5.1. Interplay of Alzheimer’s Disease and Cerebral CYPs

Alzheimer’s disease (AD) is the primary cause of dementia, with an estimated incidence of 1700–2900 cases per 100,000 individuals in the USA [266] and projections suggesting that affected individuals may reach 152 million by 2050 [266,267,268]. Between 1990 and 2019, both the incidence and prevalence of AD increased by approximately 147.95% [267,269]. AD is characterized by progressive memory loss, cognitive impairment, and behavioral disturbances, and its pathogenesis involves complex genetic and environmental factors leading to neuronal dysfunction and loss in key brain regions such as the hippocampus and neocortex. A dual proteinopathy marked by tau neurofibrillary tangles and extracellular Aβ plaques is central to the disease, with mechanisms including cholinergic nerve damage, Aβ toxicity, abnormal tau phosphorylation, and oxidative stress contributing to its progression [270].

In this context, two CYP-mediated mechanisms are particularly relevant to AD: cholesterol homeostasis and the generation of epoxyeicosatrienoic acids (EETs), as previously discussed in Section 4.1.2 and Section 4.1.4 (Figure 4). Genetic variations in CYP46A1 are associated with an increased risk of developing AD [271]. As the principal enzyme responsible for cholesterol elimination in the CNS, CYP46A1 converts cholesterol into 24-hydroxycholesterol (24-HC). Disruption of CYP46A1 function leads to an accumulation of cholesterol esters within neurons [47,58,77], a phenomenon linked to memory impairments and adverse effects in AD. Inhibition of CYP46A1 results in elevated cholesterol levels in hippocampal neurons and lipid rafts, while simultaneously reducing 24-HC levels [42]. This imbalance between cholesterol synthesis and clearance favors the formation of cholesterol ester deposits that promote the amyloidogenic processing of the amyloid precursor protein (APP) into Aβ, a hallmark of AD pathology [38,40,42,43,44,131]. Consequently, these events lead to increased APP recruitment, higher Aβ production, neuronal loss in the hippocampus, and cognitive deficits. Importantly, enhancing CYP46A1 activity can reduce cholesterol ester accumulation, thereby mitigating both Aβ and tau pathology in AD neurons derived from induced pluripotent stem cells [42,43,44]. Thus, clarifying the role of CYP46A1 in cholesterol metabolism is essential for understanding the disrupted cholesterol homeostasis underlying neurodegenerative processes.

In AD models, reduced expression and activity of CYP46A1 correlate with lower levels of the enzyme and its metabolites in the hippocampus [43]. Restoration of CYP46A1 through adeno-associated vector delivery into the hippocampus normalizes enzyme levels, reduces amyloid plaque formation, and fully restores memory function [43]. A range of endogenous substrates and pharmacological agents, including L-glutamate, L-aspartate, γ-aminobutyric acid, acetylcholine, N-methyl-D-aspartate, efavirenz, acetaminophen, and mirtazapine, have been identified as selective inducers of CYP46A1 [44,76]. Among these, L-glutamate increases enzyme activity in vitro by three-fold, while efavirenz, an antiretroviral medication, has emerged as a low-dose activator of CYP46A1, suggesting its potential as a novel treatment for AD [36,265,272]. Activation of CYP46A1 through enzyme overexpression or positive allosteric modulation has demonstrated neuroprotective effects in various AD mouse models [77,273,274,275,276], and efavirenz treatment in iPSC-derived AD neurons has reduced cholesterol accumulation and attenuated both Aβ and tau pathology [277]. A recent clinical trial [273] (NCT03706885) confirmed that efavirenz enhances CYP46A1 activity and brain cholesterol metabolism in early AD patients. Conversely, the selective CYP46A1 inhibitor soticlestat has shown significant anti-seizure effects in preclinical and clinical settings, underscoring the therapeutic potential of modulating CYP46A1 activity [278].

Recent research has also revealed sex-specific differences in the regulation of CYP46A1 [131]. A study has reported higher levels of CYP46A1 in young, cognitively normal women compared to men, suggesting a modulatory role for sex hormones. In aged mice, overexpression of CYP46A1 increases 24-HC levels and modulates neuroactive steroid signaling in a sex-dependent manner, conferring neuroprotective effects exclusively in females [279]. In aged female mice, CYP46A1 overexpression enhances estrogen signaling within the hippocampus, leading to improved cognitive functions, whereas in males, it results in anxiety-like behavior, memory impairment, and elevated 5α-dihydrotestosterone levels. These sex-specific effects, including protection from memory impairments induced by ovariectomy in females, highlight the translational relevance of CYP46A1 regulation in human AD. Clinically, CYP46A1 is particularly significant since early menopause—a female-specific risk factor—is associated with cognitive decline and exacerbated AD pathology. Therefore, understanding the sex-specific regulation of CYP46A1 is critical for developing targeted therapeutic strategies.

CYP-derived EETs are considered protective for mitochondrial dynamics; however, Aβ impairs epoxygenase activity, reducing the production of specific EETs (e.g., 11,12-EET and 14,15-EET) [168,280]. Inhibiting soluble epoxide hydrolase to elevate EET levels shows promise as a therapeutic strategy, while Aβ-induced reductions in CYP epoxygenase activity further exacerbate AD pathology [168,280]. These findings underscore that diminished CYP46A1 or CYP epoxygenase activity disrupts the balance of cholesterol and EETs (Figure 4), contributing to AD progression. Efforts to evaluate plasma 24-HC as an indicator of CYP46A1 activity have yielded inconsistent results, with some studies reporting elevated 24-HC in patients with mild cognitive impairment or early AD, and others reporting no significant differences compared to healthy controls [54,55,57,281].

### 5.2. Interplay of Parkinson’s Disease and Cerebral CYPs

Parkinson’s disease (PD) is the second most prevalent neurodegenerative disorder and is characterized by progressive motor symptoms that develop over time. Recent years have seen a rapid increase in PD’s prevalence and disability, making it a major contributor to global disability rates [282]. The Global Burden of Disease study reported 1.02 million new cases of PD in 2017 and a total of 6.1 million PD patients globally in 2016 [268,283], with the age-standardized prevalence rate rising by 21.7% from 1990 to 2016 [284]. Projections suggest a significant rise in PD cases, with an estimated 4.94 million patients in China by 2030 [282].

PD is primarily marked by the gradual depletion and progressive loss of dopaminergic neurons in the substantia nigra pars compacta, which leads to secondary dysfunction in the basal ganglia of the midbrain. This neuronal loss results in a marked reduction in dopamine levels in the striatum, a critical factor in the onset and progression of PD [46], underscoring the pivotal role of cerebral CYPs in modulating dopamine metabolism, as discussed in Section 4.1.2 (Figure 5A). In addition, PD pathology is characterized by the accumulation of fibrillar α-synuclein in various neuronal structures, which serves as a key cellular hallmark of the disease [285]. The onset of PD involves a complex interplay of neuroinflammation, mitochondrial dysfunction, oxidative stress, and environmental factors that collectively increase the vulnerability of dopaminergic neurons [286,287]. In familial cases, genetic mutations in the alpha-synuclein gene and leucine-rich repeat kinase 2 gene further increase susceptibility to PD [288].

The involvement of cerebral CYP enzymes in PD encompasses several isoforms, with CYP2D6 being particularly significant due to its predominant expression in dopaminergic neurons. CYP2D6 contributes not only to dopamine synthesis but also to the metabolism and clearance of neurotoxins [90,96]. Reduced CYP2D activity, as seen in poor metabolizers, has been associated with an increased risk of developing PD [34,289,290]. As the disease progresses, there is a notable decline in CYP2D6 protein expression—approximately a 40% reduction in PD patients [91]. Studies on mouse Cyp2d22, a homolog of human CYP2D6, demonstrate that exposure to the neurotoxin 1-methyl-4-phenyl-1,2,3,6-tetrahydropyridine (MPTP) leads to decreased mRNA expression in nigrostriatal tissues, whereas inducers such as nicotine can upregulate Cyp2d22, potentially offering neuroprotection [291,292]. Moreover, CYP2D6 has been shown to deactivate MPTP and its toxic metabolite 1-methyl-4-phenylpyridinium (MPP+), demonstrating its capacity to neutralize neuroactive substances [293]. Investigations using humanized CYP2D6-expressing transgenic mice suggest that enhanced CYP2D6 activity can protect against beta-carboline-induced behavioral neurotoxicity, indicating a potential safeguard against neurotoxicity and neurodegenerative processes [34,107,294]. However, complexities arise with mitochondria-targeted CYP2D6, which may exhibit a dual role by contributing to both neurotoxic substance production and neuroprotection through dopamine-related pathways [295,296]. This dichotomy underscores the current ambiguity surrounding the direct relationship between cerebral CYP activity changes and PD onset.

Conversely, the role of CYP2E1 in PD remains complex. Although the association between CYP2E1 polymorphisms and PD is ambiguous, CYP2E1 is expressed in the substantia nigra, and an intron 7 polymorphism in the CYP2E1 gene has been linked to PD in specific European populations [297]. The detrimental effects of increased CYP2E1 expression appear to outweigh its neuroprotective functions, as elevated CYP2E1 levels in PD are associated with increased ROS production and MPTP toxicity [45,298,299]. Furthermore, reduced methylation of CYP2E1 in PD patient brains leads to higher mRNA expression, suggesting that epigenetic regulation plays a role in PD susceptibility [300]. On another front, caffeine metabolism via CYP1A2 exhibits an inverse correlation with PD risk, hinting at a potential protective mechanism [301], while the absence of CYP19, which produces the neuroprotective steroid 17β-estradiol, is considered a risk factor for PD [302]. Overall, understanding these intricate relationships between cerebral CYP enzyme activity and PD is essential for unraveling disease mechanisms and developing targeted therapeutic strategies, including personalized medicine approaches to improve PD management.

#### 5.2.1. Interrelation Between Smoking and Parkinson’s Disease

In recent years, several studies have indicated that smoking or nicotine intake in any form reduces the risk of PD, suggesting a potential disease-modifying effect of nicotine [230,243,244,245]. Epidemiological evidence consistently shows an inverse correlation between tobacco use and PD incidence; smokers are less likely to develop Parkinson’s than non-smokers [246,249,250,303]. Nicotine has demonstrated potential benefits on hyperkinetic movements, as evidenced by both investigational studies and clinical trials. Experimental models indicate that nicotine can elevate antiapoptotic protein levels, enhance CYP enzyme activity, and provide protection against nigrostriatal pathway degeneration [245,246,247,248,249,250,303]. Additionally, cerebral CYP enzymes play a role in both deactivating nicotine (Figure 7A) and activating carcinogenic compounds present in tobacco, such as 4-[methylnitrosamino]-1-(3-pyridyl)-1-butanone and 3-(1-nitrosopyrrolidin-2-yl)-pyridine [220]. Although increased CYP enzyme levels, either due to genetic predisposition or nicotine-induced upregulation, have been linked to neural oxidative stress, they may also facilitate the detoxification of neurotoxic compounds entering dopaminergic neurons via the dopamine transporter, thereby mitigating oxidative stress, mitochondrial dysfunction, and subsequent cell death. Cerebral CYP enzymes, located within dopaminergic neurons of the substantia nigra and striatal terminals, play a vital role in metabolizing substances relevant to PD.

Smoking has been extensively studied for its protective effect against PD, with research showing that smokers have a significantly reduced risk of developing the disease—a benefit that correlates with the intensity of smoking [304]. This protective association is speculated to be partly due to a “risk-averse personality” trait observed in individuals predisposed to PD [246]. Studies on nicotine have predominantly focused on its symptomatic effects using transdermal patches [247]. For instance, a study involving 30,000 male physicians in the British Doctors cohort reported lower PD-related mortality rates in current smokers compared to never-smokers [243], and an inverse correlation was found between tobacco consumption and PD risk. However, the protective effect diminishes over time after quitting smoking.

A recent trial assessing the effects of transdermal nicotine on motor and nonmotor symptoms in early-stage PD did not demonstrate significant benefits in slowing disease progression or alleviating symptoms [246]. Similarly, a nationwide cohort study in Korea found no significant correlation between smoking status and all-cause mortality in PD patients, although smokers experienced fewer neurological-related deaths at the expense of higher risks for smoking-related cancers [249]. While the outcomes of nicotine-based therapies in PD present conflicting evidence, it remains imperative to investigate their potential benefits. The observed link between cerebral CYP-mediated nicotine metabolism and PD warrants further exploration to elucidate the underlying biological mechanisms. However, until robust evidence supports their efficacy, promoting nicotine as a treatment for PD is not advisable. This pursuit holds promise in fostering the development of targeted and efficacious therapeutic modalities in the foreseeable future.

### 5.3. Interplay of Huntington’s Disease and Cerebral CYPs

Huntington’s disease (HD) is an inherited neurodegenerative disorder characterized by an autosomal dominant mutation involving an abnormal expansion of CAG trinucleotide repeats in the huntingtin (HTT) gene, resulting in an expanded polyglutamine tract in the HTT protein [49,305]. The reported incidence of HD ranges from 0.38 to 2.71 per 100,000 persons, with an average prevalence of approximately 4.88 per 100,000 individuals, and higher rates observed in Europe and North America compared to Asia and Africa [306,307]. Clinically, HD manifests as a progressive deterioration of striatal medium spiny neurons and cortical neurons projecting to the striatum, leading to motor deficits, cognitive decline, and psychiatric disturbances, often emerging with extrapyramidal signs such as chorea, dystonia, and bradykinesia [48,49,305].

In HD, disruptions in cholesterol metabolism parallel those observed in AD. Reduced expression of CYP46A1 in the striatum, observed across various species, is associated with multiple microglial dysfunctions [47,308]. Mouse studies reveal that knockdown of CYP46A1 in the striatum results in neurodegeneration, while overexpression of CYP46A1 reverses disease pathology in HD models such as R6/2 and zQ175 [47,49,305]. Moreover, mHTT aggregates are less prevalent in astrocytes than in neurons, potentially due to more efficient degradation mechanisms in astrocytes. Astrocytes are crucial for cholesterol metabolism in the brain (Figure 4A), and HD astrocyte cultures exhibit reduced mRNA levels for cholesterol biosynthesis genes, such as CYP51, and diminished APOE protein levels [309,310]. Transcriptional dysregulation in HD astrocytes, particularly in those expressing exon1-mHTT, underscores the specific disruption of cholesterol pathways [311].

The role of CYP46A1 in cholesterol turnover and neuronal function is further demonstrated in mouse models. In CYP46A1-deficient mice, despite unchanged brain cholesterol levels, cholesterol synthesis decreases by 40%, possibly compensating for the lack of degradation, resulting in severe cognitive deficits and impaired synaptic plasticity [125]. Conversely, transgenic mice overexpressing human CYP46A1 (C46-HA) show increased 24-HC production, enhanced cholesterol synthesis (evidenced by elevated lanosterol levels), improved spatial memory, and higher levels of neurotransmission-related proteins [312]. In vitro studies indicate that increased CYP46A1 levels promote dendritic growth and enhance synaptic protein expression, highlighting its critical role in synaptic function [313].

In HD patients, CYP46A1 expression is reduced in the postmortem putamen and in the striatum of HD mouse models such as R6/2 and zQ175 [47,49,305,308]. Adeno-associated virus-mediated knockdown of CYP46A1 in the striatum of wild-type mice replicates HD-like neurodegeneration and motor deficits [47]. Recent gene therapy approaches delivering CYP46A1 into the striatum of HD models have improved neuronal atrophy, reduced mHTT aggregates, and enhanced motor behavior, accompanied by boosted cholesterol metabolism—increased 24-HC production, elevated cholesterol precursor levels, and upregulated cholesterogenic enzyme expression [47,49,305,308]. These therapies also induced a broad transcriptomic shift in HD zQ175 mice, improving synaptic transmission, vesicular transport, and protein metabolism pathways. Additional studies in neuroblastoma models of HD demonstrate that CYP46A1 overexpression reduces both the quantity and size of mHTT aggregates, potentially via autophagy activation, and protects against NMDA-mediated excitotoxicity [47,48]. Given the promising results from CYP46A1-based gene therapy, particularly with recent advancements in vector delivery strategies [314], further research is warranted. However, the interpretation of plasma 24-HC as a biomarker for brain cholesterol metabolism remains challenging due to its susceptibility to liver metabolism. Recently, a noninvasive CYP46A1-targeted PET tracer (18F-Cholestify) has emerged, offering precise quantification of CYP46A1 abundance and cholesterol metabolism across various brain regions in rodents, nonhuman primates, and humans, thereby providing valuable insights into cholesterol homeostasis in HD and other conditions [131]. A deeper exploration of the intricate interplay between cerebral CYP enzyme function and HD is vital for advancing our insights and refining treatment approaches.

### 5.4. Interplay of Neuropsychiatric Disorders and Cerebral CYPs

Neurological disorders that manifest with psychiatric symptoms arise from a complex interplay of biological, psychological, and environmental factors. In this context, cerebral CYP enzymes exert a multifaceted influence by modulating the metabolism of neuroactive compounds (Figure 7B) and regulating cholesterol homeostasis (Figure 4A). The impact of psychiatric disorders on cerebral CYP-mediated metabolism differs from that observed in neurodegenerative diseases. In psychiatric conditions, heightened activity of specific CYP isoforms in distinct brain regions can lead to imbalances in neurotransmitter levels—particularly within critical monoamine systems such as dopamine, serotonin, and norepinephrine—which, in turn, profoundly affect behavior, cognition, and emotional regulation. Moreover, impaired cholesterol metabolism in the brain, as reflected by fluctuations in 24-HC levels governed by CYP46A1 activity, further underscores the pivotal role of cerebral CYPs across a broad spectrum of neurological and psychiatric disorders. This dual impact on both cholesterol metabolism and neurotransmitter regulation highlights the extensive influence of cerebral CYP enzymes in maintaining normal brain function.

Initially identified as potential therapeutic targets primarily in major neurodegenerative conditions such as AD, PD, and HD, cerebral CYP enzymes have since been recognized for their regulatory roles in various neuropsychiatric disorders. These disorders include, but are not limited to, schizophrenia [50,51,315,316], depression [316,317,318,319], anxiety [320,321], epilepsy [128], autism [54,322], bipolar disorder [54], Niemann–Pick disease type C [137], and spinocerebellar ataxia [323], among others [36,138,279,324].

Schizophrenia (SCZ), a severe psychiatric disorder with a strong genetic basis, affects approximately 1% of the population and significantly impacts quality of life and mortality, often leading to permanent brain damage. SCZ is characterized by positive symptoms such as hallucinations, negative symptoms like social withdrawal, and cognitive impairments, including memory deficits. Globally, SCZ affects roughly 24 million people—about 1 in 300 individuals—with lifetime occurrence rates ranging from 3.8% to 8.4% [325,326]. The raw prevalence of schizophrenia is estimated between 14.2 and 23.6 million, with incidences ranging from 941,000 to 1.3 million and disability-adjusted life years (DALYs) between 9.1 and 15.1 million, all showing significant increases [327]. Despite pharmacological interventions, SCZ progression remains a major concern due to the substantial variability in the efficacy of antipsychotic medications, with up to fifty percent of patients experiencing suboptimal symptom relief or adverse drug reactions.

Elevated CYP2D6 expression in brain neurons, particularly in the hippocampus, plays a significant role in dopamine transformation and neurotransmission, potentially influencing susceptibility to SCZ. One core mechanism in SCZ pathogenesis is hippocampal hyperactivity, which leads to heightened dopamine neuron activity and increased responsiveness to dopamine [316]. CYP2D6, co-localized with the dopamine transporter in dopaminergic neuron membranes, not only participates in dopamine transmission but also converts dopamine from tyramine—a process that underscores its intertwined effects on the central dopaminergic system (Figure 5A). The association of CYP2D6 with SCZ has been further explored through the identification of specific single-nucleotide variants (e.g., rs16947 and rs133377), which correlate with increased exon 3 skipping and reside within active transcription start sites, as demonstrated by histone acetylation analysis [50,328]. Furthermore, CYP2D6 plays a pivotal role in metabolizing psychiatric medications such as aripiprazole and risperidone, impacting both plasma drug levels and clinical outcomes in SCZ [50]. Additionally, CYP2D6 is involved in the metabolism of neuroactive steroids implicated in both SCZ and bipolar disorder.

A recent study investigating the interconnections among CYP2D6 genotype, hippocampal white matter integrity, and antipsychotic treatment response in Korean SCZ patients found that intermediate metabolizers exhibited higher fractional anisotropy and lower radial diffusivity in the right hippocampus compared to extensive metabolizers. Moreover, following antipsychotic treatment, extensive metabolizers showed greater improvements in positive symptoms, correlating with these imaging changes [51]. In a separate study of 691 European-ancestry patients with severe mental illness, a notable correlation was found between CYP2D6 activity scores and symptom severity, with higher metabolic activity associated with increased positive symptoms and cognitive decline, particularly in patients with more severe manifestations [52].

Ultimately, cerebral CYP enzymes play a pivotal role in regulating cholesterol homeostasis, dopamine metabolism, and neurotransmitter dynamics, significantly influencing the pathophysiology of both neurodegenerative and neuropsychiatric disorders. By deepening our understanding of brain CYP metabolism, these insights offer promising avenues for developing targeted therapeutic strategies to improve clinical outcomes and support neurological health.

## 6. Emergence of CYP-Based Neuropathological Biomarkers

Neurological disorders affect approximately 50 million individuals worldwide, yet therapies capable of modifying their progression remain limited. Decades of neuropathological research combined with advances in biomedical advances have unraveled complex, interconnected mechanisms underlying neurodegeneration. Although postmortem examinations remain the definitive diagnostic standard, their reliance on cross-sectional histopathological assessments limits the ability to monitor dynamic, temporal changes. Consequently, researchers increasingly turn to antemortem biomarkers to monitor disease evolution across stages, even when these findings do not fully align with postmortem observations [329].

A central limitation of traditional neuropathology is its focus on end-stage disease, which may obscure early pathophysiological changes critical for effective intervention. In this context, cerebral CYP enzymes have emerged as promising biomarkers. As discussed earlier, dysregulation of CYP enzymes is closely linked with neuroinflammation, oxidative stress, and neuronal dysfunction, offering substantial potential for both diagnostic and prognostic applications. This section presents an integrated overview of CYP-based biomarkers, emphasizing their role in understanding disease mechanisms and advancing precision medicine in neurological care.

The rationale for exploring CYP-based biomarkers lies in their essential contribution to neurological homeostasis. Alterations in CYP expression, enzymatic activity, or regulatory mechanisms can serve as early indicators of neurodegeneration, offering insights into disease progression and potential therapeutic targets. Moreover, the susceptibility of CYP enzymes to epigenetic modifications reinforces their utility as biomarkers. CYP-based biomarkers are broadly categorized as follows: (i) Genetic biomarkers: Variants or polymorphisms in CYP genes have been linked to a predisposition to neurological disorders, supporting early risk assessment and personalized treatment strategies. (ii) Epigenetic biomarkers: Epigenetic modifications, such as DNA methylation and histone alterations in CYP genes, reflect changes in gene expression and enzyme activity, offering insights into disease mechanisms and intervention points. (iii) Metabolic biomarkers: Metabolites generated via CYP-mediated pathways, including lipid metabolites and neuroactive compounds, can be quantified in biofluids (e.g., CSF or plasma) to enhance diagnostic precision and monitor disease progression.

In AD, traditional laboratory diagnosis relies on CSF biomarkers—amyloid-β42 (Aβ42), total tau, and phospho-tau. However, these methods are often invasive and expensive, warranting alternative diagnostic and therapeutic strategies. Recent investigations have focused on CYP-based mechanisms in AD progression. For example, a recent study analyzed DNA methylation changes in CYP genes using circulating free DNA (cfDNA) from 25 AD patients and 23 healthy controls [270]. Utilizing logistic regression via MetaboAnalyst and pathway analysis with Cytoscape, the study identified disruptions in androgen/estrogen biosynthesis, C21 steroid hormone metabolism, and arachidonic acid metabolism, with the CYP-based cfDNA biomarkers achieving an area under the ROC curve (AUC) of 0.942 (95% CI: 0.905–0.979) and 90% sensitivity and specificity [270]. Another investigation reported distinctive alterations in CYP/soluble epoxide hydrolase (sEH) and acylethanolamide metabolism in both plasma and CSF [330]. Robust predictive and discriminant models suggest their potential as AD-associated metabolic disruption biomarkers. This reinforces that a combined strategy—inhibiting sEH while enhancing acylethanolamide levels—may offer a novel therapeutic approach for AD, compared to targeting each pathway separately.

A focused discussion on CYP46A1-mediated cholesterol metabolites highlights their direct diagnostic and therapeutic potential. Specifically, 24-hydroxycholesterol (24-HC) and 27-hydroxycholesterol (27-HC) serve as surrogate biomarkers for mild cognitive impairment (MCI) and AD progression [135]. Neuronal cholesterol elimination via 24-HC affects amyloid precursor protein processing and is associated with brain atrophy and gray matter loss, with CSF levels correlating with AD markers such as Aβ, tau, and phospho-tau [331]. Quantifying CSF or plasma levels of 24-HC provides a direct measure of neuronal cholesterol elimination. In parallel, 27-HC, which crosses the BBB, contributes to amyloid deposition, thereby linking hypercholesterolemia with AD pathogenesis [135]. Moreover, recent findings indicate that CYP46A1 activation exerts sex-specific effects; in aged mice, CYP46A1 overexpression increased 24-HC levels and modulated neuroactive steroid signaling—providing neuroprotection by reducing total tau levels specifically in females [279]. Together with traditional CSF markers, these biomarkers offer a sensitive means to evaluate both MCI and AD. Therapeutically, modulating CYP46A1 activity to optimize these metabolite levels could offer a dual strategy—enhancing diagnostic accuracy while opening avenues for targeted intervention.

Vitamin D metabolism, long recognized for its role in calcium homeostasis, is also critical for brain health and is implicated in neurodegenerative disorders such as AD, PD, and Multiple-System Atrophy (MSA). Recent studies have linked altered levels of vitamin D metabolites with changes in CYP activity. Dysregulation in these enzymatic processes not only alters vitamin D status but also contributes to neurological deficits. The conversion of 7-dehydrocholesterol to 25-hydroxycholecalciferol (25(OH)D) and then to its active form, 1,25-dihydroxyvitamin D (1,25(OH)_2_D), involves enzymes such as microsomal CYP2R1 and mitochondrial CYP27B1 [332]. Deficiencies in these metabolites correlate with neurodegeneration, and key brain regions like the hypothalamus and substantia nigra prominently express vitamin D receptors and activating enzymes. The study also compared serum levels of 25(OH)D and 1,25(OH)_2_D among healthy subjects, MSA, and PD patients, revealing that MSA individuals had lower levels of both biomarkers, whereas PD patients primarily exhibited reduced 25(OH)D [332]. These findings, together with associations between biomarker levels and clinical severity, underscore their potential in distinguishing neurodegenerative conditions. Another investigation explored the Mini-Mental State Examination (MMSE) and reported that the serum 25(OH)D_3_ and 1,25(OH)_2_D_3_ levels provided insights into reduced serum 25-hydroxyvitamin D levels in both AD and PD patients, particularly among ApoEε4 noncarriers, potentially influenced by CYP27A1 affecting vitamin D synthesis [333,334]. These findings underscore the interplay between CYP-mediated vitamin D metabolism and neurodegeneration, suggesting that vitamin D biomarkers can be integrated with CYP-based markers to form a more comprehensive diagnostic panel [332,333,334].

Transitioning from metabolic biomarkers to inflammatory processes, it is important to recognize that cytokines such as interleukin-1β, interleukin-6, and tumor necrosis factor α modulate CYP enzyme expression via nuclear factor-κB signaling [335]. In PD, for example, inflammation-induced changes in CYP expression are observed both in the brain and peripheral tissues like the liver, correlating with dopaminergic neuron degeneration [41,300]. To bridge these areas, a novel assay utilizing recombinant CYP enzymes expressed on bacterial membranes—with serum and the fluorescent substrate Vivid^®^—has been developed to assess CYP-mediated oxidation [336]. This assay effectively distinguishes PD samples from healthy controls with high AUC values in both preclinical and clinical studies, thereby integrating inflammatory and metabolic biomarkers for improved diagnostic specificity.

Despite their promising potential, the clinical implementation of CYP-based biomarkers faces several challenges, including the need for standardized assay protocols, managing inter-individual variability due to genetic and environmental factors, and addressing the invasive nature of sample collection (e.g., reliance on CSF). Moreover, robust validation in diverse patient cohorts is necessary to fully integrate these biomarkers with existing diagnostic modalities. However, recent advances in mass spectrometry, next-generation sequencing, and bioinformatics have accelerated the discovery and validation of CYP-related molecular signatures, with high-throughput omics approaches enabling comprehensive profiling that deepens our understanding of neuropathological mechanisms and paves the way for precision medicine. The integration of CYP-based biomarker panels with traditional diagnostic tools holds significant promise for early disease detection, improved prognostic assessments, and the development of targeted therapeutic interventions. Collaborative, interdisciplinary efforts will be essential to overcome current implementation challenges and to fully harness the transformative potential of CYP-based neuropathological biomarkers in clinical practice.

## 7. Future Perspectives in Brain CYPs: Emerging Insights and Therapeutic Opportunities

The future of neuroscience is poised on the brink of groundbreaking discoveries, with cerebral CYP enzymes emerging as pivotal players in the intricate landscape of brain function and neurological health. Cerebral CYP enzymes have been found to play significant roles in neurotransmitter dynamics, neuroinflammation, and neurosteroid metabolism, offering new insights into how the brain metabolizes endogenous substances and how this process is implicated in neurological disorders. As our understanding of cerebral CYPs deepens, it opens up several avenues for future research.

One particularly compelling area is the relationship between cerebral CYP enzymes and neurodegenerative diseases. Modulating CYP activity may mitigate disease progression, while their influence on cognitive processes such as learning, memory, and behavior underscores their broader significance in maintaining brain health. The development of brain-specific CYP inhibitors, along with their validation in animal models, represents a crucial step towards more precise therapeutic strategies, enabling researchers to refine their approaches in neuroscientific investigations. In vivo studies such as brain microdialysis and neuroimaging will be indispensable for elucidating the metabolic processes mediated by cerebral CYPs, facilitating the identification of neuropathological biomarkers. This, in turn, could lead to advancements in precision medicine, improving the care and treatment of neurological conditions.

Recent advances in artificial intelligence (AI) have significantly enhanced our capacity to predict the functions of cerebral CYP enzymes and their roles in neurodegenerative diseases [337,338,339]. Deep learning models such as AlphaFold provide highly accurate predictions of CYP enzyme structures and active sites, thereby facilitating detailed modeling of substrate interactions [340]. In the realm of Alzheimer’s precision neurology, the integration of omics, high-throughput technologies, and computational approaches has enabled the identification of novel system dysfunctions and the development of precise biomarkers [270]. By incorporating diverse data types, AI can uncover potential disease pathways and predictive biomarkers in neurodegeneration. For example, one study leveraged the Parkinson’s Progression Markers Initiative (PPMI) database to extract gene sequences of all 57 human CYP enzymes and their redox partners, analyzing the association of single-nucleotide polymorphisms (SNPs) with PD [341]. This comprehensive “CYPome-wide” analysis revealed that SNPs in 26 of the 57 CYP enzymes were at least five-fold over-represented in PD patients, implicating these CYPs as potential players in the pathogenesis of PD. Moreover, AI-driven analysis of metabolite profiles offers promising avenues for improving PD diagnosis. For instance, the combination of a CYP inhibition assay with AI algorithms has been used to differentiate PD patients from healthy controls by detecting alterations in metabolite levels associated with cerebral CYP activity [342]. These metabolite changes, reflecting the impact of inflammation and exposure to endogenous or exogenous substances on CYPs (as discussed in Section 4), provide a distinctive diagnostic signature. Machine learning models further analyze these complex metabolite datasets to identify CYP-mediated metabolic pathways and informative diagnostic biomarkers [343], thereby enabling earlier and more accurate ND detection. Beyond diagnostics, AI also facilitates the development of therapeutic interventions; molecular docking and 3D Quantitative Structure–Activity Relationship models can predict the CYP inhibitory activities of candidate compounds, ultimately identifying potential therapeutic agents for PD treatment [342].

Another promising research area focuses on the transmembrane domains of brain CYP enzymes, which are essential for their proper localization within cellular membranes [17]. To enhance clarity, this complex topic can be divided into two parts: first, the transmembrane segments ensure that CYP enzymes are accurately anchored within the lipid bilayer; second, these domains modulate the enzymes’ interactions with substrates and regulatory molecules, influencing substrate access and governing interactions with hydrophobic compounds and the BBB [10]. Understanding how brain CYPs metabolize hydrophobic compounds and interact with the BBB is crucial for elucidating their roles in central nervous system physiology and pathology.

Further, exploring the structural differences between brain and hepatic CYPs offers intriguing opportunities for further investigation. By exploring these differences, researchers can gain insights into the distinct metabolic profiles and substrate preferences of these enzymes. Advanced analytical techniques, such as mass spectrometry and live-cell NMR, are particularly suited to this task, enabling comprehensive analyses of the metabolic pathways governed by brain CYPs. Understanding why specific CYP isomers function differently in the brain and liver is key to deciphering the varying metabolic outcomes observed in CNS disorders.

Intriguingly, beyond their neuroprotective roles, CYP enzymes emerge as critical regulators of cardiometabolic health, particularly in regulating vascular homeostasis. They are key players in the pathogenesis of cardiovascular diseases such as ischemic and hemorrhagic strokes, as well as traumatic brain injury (TBI) [344,345,346]. As discussed in Section 4.1.4, CYP enzymes metabolize fatty acids like AA into bioactive eicosanoids, including EETs and HETEs (Figure 4B). These metabolites play essential roles in regulating vascular tone and inflammation, with EETs promoting vasodilation and neuroprotection, while HETEs induce vasoconstriction under pathological conditions [347]. Recent studies have illustrated that genetic variants in CYP genes significantly influence individual responses to cardiovascular risk factors. For example, CYP2C19 polymorphisms alter the metabolism of clopidogrel, a critical antiplatelet drug, impacting its efficacy in preventing ischemic events [348]. CYP4A, which produces HETEs, has been linked to vasoconstriction, hypertension, and cerebral hemorrhage risk [349]. In TBI, CYP enzymes regulate metabolic responses and inflammation, producing neuroprotective eicosanoids [350]. Understanding the genetic and functional dynamics of CYP enzymes can guide personalized treatments for stroke and TBI, improving therapeutic strategies in cardiometabolic health.

Finally, the intrinsic flexibility of brain CYPs, which influences their enzymatic function and substrate specificity, is a noteworthy characteristic that warrants further study. The formation of hetero-oligomers and mono-oligomers among brain CYPs suggests synergistic interactions that could modulate metabolic pathways and fine-tune neuronal responses. Leveraging these insights into brain CYPs and their interactions with the CNS microenvironment holds immense potential for the development of targeted therapeutic interventions. By harnessing the synergistic properties and inherent flexibility of brain CYPs, researchers are well positioned to pioneer innovative and tailored treatment approaches that could significantly improve patient outcomes in neurology and beyond. Collectively, these research directions underscore the critical need for comprehensive investigations into human cerebral CYPs, their regulation, and their metabolic roles in both health and disease. As these mechanisms are further elucidated, cerebral CYP enzymes are expected to become central to innovative, tailored treatment strategies for complex neurological disorders while reinforcing the scientific rigor and translational potential of this research.

## 8. Conclusions

The evolutionary trajectory of brain cytochrome P450 enzymes underscores their indispensable role in regulating metabolic processes critical to neurological health and disease. Cerebral CYPs are not only central to neurotransmitter regulation, neuroinflammation, and neurosteroid metabolism but also mediate the detoxification of neurotoxins and the biotransformation of drugs, functions essential for maintaining central homeostasis. Dysregulation of cerebral CYPs has tangible impacts on disease progression, as accumulating evidence increasingly positions the potential of these enzymes as both biomarkers and therapeutic targets. For instance, reduced CYP46A1 activity has been linked to amyloid-beta accumulation and cognitive decline in AD, while altered CYP2D6 expression in the basal ganglia correlates with increased susceptibility to PD. Furthermore, the up- or downregulation of various CYPs can elevate ROS, triggering neuroinflammation and contributing to neurotoxic effects.

As ongoing investigations continue to unravel the intricate interplay between cerebral CYPs and metabolic pathways, they hold the promise of revolutionizing our understanding of brain physiology, pathology, and pharmacology, ultimately leading to more effective and tailored treatment paradigms for neurological health. Moreover, the structural and functional flexibility of cerebral CYPs not only enhances our comprehension of brain metabolism but also lays the groundwork for optimizing neuropharmacotherapy outcomes. Advancements in biomedical sciences, especially AI-driven structural modeling, multi-omics analyses, and integrated neuroimaging and metabolomic profiling, are transforming our ability to predict brain CYP functions and interactions. These innovative tools enable precise mapping of CYP-mediated metabolic pathways and facilitate the identification of novel biomarkers, thus paving the way for precision medicine. Concurrently, the development of brain-specific CYP inhibitors and comprehensive characterization of CYP polymorphisms hold promise for personalized therapeutic interventions with minimal systemic side effects.

Cerebral CYPs play crucial roles beyond conventional metabolic functions, engaging in crucial processes that could dictate neurodegenerative outcomes. As we unravel the complexities of CYP-mediated metabolism, emerging technologies will enhance our understanding of brain health and improve therapeutic strategies. The pursuit of knowledge in this field may not only lead to effective treatments for neurodegenerative diseases but also unlock new paradigms in neurological care. By tailoring treatments to individual metabolic profiles, we stand at the threshold of a new era in personalized medicine for neurological disorders. The future of brain CYP research is promising and ripe with potential, holding the key to significant advancements in combating neurodegenerative disorders. As we deepen our exploration and understand these complex enzymes, we move closer to transformative breakthroughs in neurological health and treatment.

## Data Availability

The original contributions presented in this study are included in the article. Further inquiries can be directed to the corresponding authors.

## Abbreviations

The following abbreviations are used in this manuscript:

CYP	Cytochrome P450
CNS	Central nervous system
CSF	Cerebrospinal fluid
BBB	Blood–brain barrier
AD	Alzheimer’s disease
PD	Parkinson’s disease
HD	Huntington disease
SCZ	Schizophrenia
ROS	Reactive oxygen species
LXR	Liver X receptor
NMDAR	N-methyl-D-aspartate receptor
APP	Amyloid precursor protein
Aβ	Amyloid β
24-HC	24S-hydroxycholesterol
5-HT	Serotonin
5-HTP	5-hydroxytryptophan
5-MT	5-methoxytryptamine
PUFA	Polyunsaturated fatty acid
AA	Arachidonic acid
EET	Epoxyeicosatrienoic acid
Ep-PUFA	Epoxy polyunsaturated fatty acid
EH	Epoxide hydrolases
LA	Linoleic acid
DHET	Dihydroxyeicosatrienoic acids
M3G	Morphine-3-glucuronide
M6G	Morphine-6-glucuronide
MPTP	1-methyl-4-phenyl-1,2,3,6-tetrahydropyridine
MPP+	1-methyl-4-phenylpyridinum
HTT	Huntingtin
mHTT	Mutant HTT
25(OH)D	25-hydroxycholecalciferol
1,25(OH)2D	25-Dihydroxyvitamin D
MSA	Multiple-System Atrophy
